# Parental Psychosocial Flourishing and Adolescents’ Charitable Giving Intention: The Roles of Adolescent Psychosocial Flourishing and Authenticity

**DOI:** 10.3390/bs16091480

**Published:** 2026-08-25

**Authors:** Huanhuan Zhao, Tianyi Ma, Yan Xu, Heyun Zhang

**Affiliations:** 1 School of Psychology, Shanghai Normal University, Shanghai 200234, China; hhzhaopsy@shnu.edu.cn (H.Z.); 1000546543@smail.shnu.edu.cn (T.M.); 2 Faculty of Psychology, Beijing Normal University, Beijing 100875, China; xuyan@bnu.edu.cn

**Keywords:** intergenerational well-being, psychosocial flourishing, charitable giving intention, authenticity, parent–adolescent dyads

## Abstract

Adolescent charitable giving (CG) is vital for philanthropic sustainability. While parental influence is recognized, the association between parental psychosocial flourishing and adolescents’ CG intention, as well as the psychological processes underlying this association, remains underexplored. Drawing on spillover–crossover theory, this research examined a moderated mediation model in which adolescents’ psychosocial flourishing mediates the link between parental psychosocial flourishing and adolescents’ CG intention, with adolescents’ authenticity moderating this pathway. Two studies were conducted with Chinese parent–adolescent dyads. Study 1 (*N* = 1179 dyads) employed a cross-sectional design to provide preliminary evidence, and Study 2 (*N* = 347 dyads) used a three-wave longitudinal design to re-examine the model with temporal ordering. Across both studies, parental psychosocial flourishing was positively associated with adolescents’ psychosocial flourishing, which in turn statistically mediated the association between parental psychosocial flourishing and adolescents’ CG intention. Moreover, adolescents’ authenticity moderated the link between adolescents’ psychosocial flourishing and CG intention, with the indirect effect of parental psychosocial flourishing on CG intention via adolescents’ psychosocial flourishing being more pronounced among adolescents with higher authenticity. These findings contribute to an intergenerational understanding of family-related antecedents in the development of adolescents’ CG intention and offer considerations for future research on family well-being and adolescent charitable engagement.

## 1. Introduction

Philanthropy serves as a voluntary mechanism for social resource redistribution (Bekkers & Wiepking, 2011a; Yang et al., 2025) and plays a vital role in promoting individual well-being (Aknin et al., 2012; Aknin et al., 2022; Nelson et al., 2016). The 2023 Global Philanthropy Tracker Report documents that 47 major economies contributed over $70 billion in charitable donations during 2020 (School of Philanthropy, Indiana University Lilly Family, 2023), reflecting broad societal engagement in prosocial resource allocation. At the individual level, charitable giving (CG) is rooted in the internalized principle of care—a fundamental moral value that motivates helping behaviors toward people in need (Bekkers & Ottoni-Wilhelm, 2016). As a core prosocial behavior, CG yields consistent psychological benefits, including enhanced positive emotions, greater happiness, and reduced depressive symptoms (Aknin et al., 2022; Y. Chen, 2023; Y. Zhang et al., 2025). Given these well-documented benefits, identifying the developmental antecedents of CG carries meaningful theoretical value and practical implications for sustainable philanthropy.

Nurturing the next generation of donors is a strategic priority for the long-term sustainability of the philanthropic sector. As the future backbone of civic society, adolescents constitute a critical population for sustaining charitable ecosystems, particularly given the developmental trajectory of prosocial behavior during this life stage. Research indicates that prosocial giving increases with age as adolescents transform from passive recipients into active contributors (Spaans et al., 2023). Consequently, understanding how to stimulate adolescents’ CG intention and enhance their willingness to engage in charitable activities has emerged as a central issue in philanthropic research. Identifying the key drivers of adolescent charitable behavior not only informs charitable education systems and cultivates socially responsible citizens but also provides theoretical foundations for the sustainable development of philanthropic societies.

Research has consistently identified the family as the micro-foundation of individual charitable behavior (Yang et al., 2019), with parents serving as primary socialization agents who profoundly shape children’s perspectives on giving (Shi, 2020). Parental emotional experiences, values, and behavioral patterns exert significant influence on adolescent development through intergenerational transmission processes (Grønhøj & Thøgersen, 2009; Leerkes et al., 2020; Li et al., 2023), to which adolescents are particularly receptive given their dependence on parental support (Pomerantz & Thompson, 2008). Although extensive research has documented factors influencing charitable behavior (Bekkers & Wiepking, 2011b; Wiepking & Bekkers, 2012), the specific pathways linking parents’ psychosocial flourishing—an indicator of optimal psychological and social functioning (Diener et al., 2010; Nelson et al., 2016; Ryff, 1989)—to adolescents’ CG intention remain underexplored from an intergenerational perspective. Notably, it remains unclear whether adolescents’ own psychosocial flourishing serves as a psychological conduit in this cross-generational association.

The spillover–crossover theory (Bakker & Demerouti, 2018) provides a framework for understanding how psychological experiences may be linked between individuals (crossover) and extended within individuals from internal experiences to behavioral tendencies (spillover). Building on this framework, we employ “crossover” and “spillover” as analytical heuristics to frame the statistical associations in our model: the association between parental and adolescent psychosocial flourishing is interpreted through a crossover lens, and that between adolescent psychosocial flourishing and CG intention through a spillover lens. It is important to emphasize that these theoretical labels denote conceptual heuristics for interpreting statistical patterns, not claims about experimentally verified interpersonal transfer or intra-individual extension processes. Complementarily, the broaden-and-build theory (Fredrickson, 2001) offers a process-oriented account, positing that positive experiences expand individuals’ thought-action repertoires and, through recurrent encounters, build enduring personal resources. In the crossover stage, parental psychosocial flourishing may facilitate family interactions that broaden adolescents’ cognitive-emotional processing, supporting the internalization of psychosocial flourishing and the cultivation of CG intentions. In the spillover stage, these accumulated resources may expand charitable cognitions and strengthen charitable inclinations (Moradi et al., 2018). Building on these theoretical foundations, the present study proposes a moderated mediation model to examine the association between parental psychosocial flourishing and adolescent CG intention, with adolescents’ psychosocial flourishing as a key mediator and authenticity—a personality trait that facilitates the alignment of internal experiences with external actions—as a moderator.

### 1.1. Intergenerational Association in Psychosocial Flourishing

Psychosocial flourishing refers to the positive and holistic development of individuals, reflecting an optimal and sustained sense of well-being (De la Fuente et al., 2020; Diener et al., 2010). Rather than merely maximizing pleasure and minimizing pain, flourishing entails the active cultivation of all life domains toward their fullest potential (Danvers et al., 2016). Conceptualized as a broad construct, it typically encompasses meaning and purpose, competence, engagement, self-acceptance, supportive relationships, optimism, concern for the well-being of others, and being respected by others (Diener et al., 2010; Ryff & Keyes, 1995; Tang et al., 2016). As the epitome of mental health, psychosocial flourishing not only represents positive functioning but also serves as an active protective factor against mental disorders (Keyes et al., 2010). Individuals with high levels of flourishing possess richer psychological resources and strengths (Diener et al., 2010). Given that adolescence represents a critical period for psychological maturation (Tian et al., 2022; Waigel & Lemos, 2023; Witten et al., 2019) and that higher well-being during this period supports adaptive development in adulthood (O’Connor et al., 2017), fostering adolescents’ psychosocial flourishing is vital for their individual welfare. However, few existing studies have examined whether parents’ psychosocial flourishing is linked to adolescents’ psychosocial flourishing from an intergenerational perspective.

Existing literature on intergenerational transmission has documented consistent associations between parents’ and their offspring’s psychological characteristics (Meeusen, 2014; Van Ijzendoorn, 1992). Intergenerational transmission refers to the familial process whereby parents’ stable cognitive dispositions, value orientations, and behavioral patterns are transferred to and internalized by their children through socialization, yielding intergenerational similarity in psychological characteristics (Meeusen, 2014; Van Ijzendoorn, 1992). This process is driven by multiple mechanisms—including genetic predispositions, shared household environments, and social learning (Augustijn, 2022; Bartels, 2015; Casas et al., 2008; Deater-Deckard et al., 2024; Grønhøj & Thøgersen, 2009)—which jointly foster congruence in psychological functioning between parents and children. Rather than testing these mechanisms, the present study focuses on the associative patterns between parental and adolescent psychosocial flourishing, aligning with the phenomenon of intergenerational similarity (i.e., parent–child similarity in psychological functioning) documented in this field.

As outlined in the theoretical framework above, this cross-generational association is framed within a crossover analytical perspective (Bakker & Demerouti, 2018). Parents with higher psychosocial flourishing tend to report more consistent and adaptive functioning across psychological, social, and emotional domains as well as warmer and more supportive parent–child relationships (Lampropoulou, 2018). Adolescents who report feeling securely cared for and valued also report higher overall functioning and psychosocial flourishing (Davis et al., 2025). This covariation is consistent with the possibility that, through sustained daily interactions, adolescents’ psychosocial flourishing parallels that of their parents. The broaden-and-build theory (Fredrickson, 2001) further clarifies how this crossover may occur: parents with high psychosocial flourishing engage in more frequent supportive interactions and model adaptive functioning, which serve as proximal environmental inputs that broaden adolescents’ momentary cognitive and attentional scope and build enduring personal and social resources over time (Fredrickson, 2001). As parents shape children’s internal working models of positive functioning through these adaptive interpersonal exchanges, adolescents gradually internalize not only the relational warmth but also the broader cognitive-behavioral repertoires associated with flourishing (Eisenberg et al., 1998; Kalbfleisch, 2024).

Emerging studies provide indirect support for this intergenerational association. The intergenerational transmission of subjective well-being—a related yet distinct construct—has been firmly established. For instance, maternal subjective well-being positively predicts children’s well-being, and parents’ life satisfaction and positive emotions are transmitted to their offspring (Carlsson et al., 2014; Casas et al., 2008; Clair, 2012). Given that psychosocial flourishing involves both hedonic and eudaimonic dimensions (Keyes, 2002; Zhao & Zhang, 2023), these findings suggest intergenerational continuity in flourishing more broadly. We therefore hypothesize:

**Hypothesis 1.**  *Parents’ psychosocial flourishing is positively associated with adolescents’ psychosocial flourishing.*

### 1.2. Psychosocial Flourishing and CG Intention

According to the theoretical framework established above, parental psychosocial flourishing may be associated with adolescents’ CG intention through two pathways: a direct “crossover” effect and an indirect effect via adolescent psychosocial flourishing. Psychological and emotional experiences can spill over across contexts and cross over between individuals (Bernardi et al., 2017; Erreygers et al., 2019). Specifically, parents with higher psychosocial flourishing tend to report a family environment characterized by positive affect and prosocial modeling, including CG exemplars. In our analytical framework, their well-being is conceptualized as crossing over to children through supportive interactions, providing adolescents with broader well-being outcomes such as positive emotions, self-esteem, meaning, and self-connectedness (Huta, 2012), which may enhance prosocial acts like CG. The happiness and love that parents exude towards their children are associated with stronger prosocial behavior in children (Erreygers et al., 2019). Conversely, children of parents with lower psychosocial flourishing tend to experience more negative emotions (Reid, 2015) and exhibit fewer prosocial behaviors (Mesurado et al., 2018). These considerations suggest a positive intergenerational link between parents’ psychosocial flourishing and adolescents’ CG intention.

Building on the spillover process described above, the pathway from adolescent psychosocial flourishing to CG intention can be understood as the intra-individual extension of internal positive functioning into charitable behavioral intentions and broader prosocial tendencies (Bakker & Demerouti, 2018). As outlined in the broaden-and-build framework, the enduring personal and social resources accumulated through positive experiences expand individuals’ cognitive and motivational scope (Fredrickson, 2001). This expanded scope allows adolescents to attend to others’ needs, recognize charitable opportunities, and construct stable charitable values over time. In other words, the resource-rich state associated with high psychosocial flourishing does not merely co-occur with transient positive states; it is associated with broader cognitive-behavioral repertoires, within which CG represents a behavioral expression of their flourishing.

Empirical evidence supports this spillover pathway. Psychosocial flourishing contributes not only to individual well-being but also to societal welfare (Moradi et al., 2018; Nelson et al., 2016), suggesting that adolescents with optimal functioning are more likely to engage in constructive contributions. Flourishers exhibit greater capability for prosocial behaviors (Moradi et al., 2018), and those in positive affective states demonstrate increased generosity and social responsibility (Isen, 2008). Key indicators of high psychosocial flourishing, such as positive emotions and autonomy, are positively linked to prosocial behaviors (Gagné, 2003; Seligman, 2011). Furthermore, flourishers tend to exhibit self-esteem, competence, and supportive interactions (Huppert & So, 2013; Seligman, 2011), which facilitate prosocial engagement (Moradi et al., 2018). Given that CG represents a typical prosocial behavior involving the voluntary sacrifice of personal resources (money, property assets, or goods) to benefit those in need (Bekkers & Wiepking, 2011b; Havens et al., 2006; C.-J. Liu & Hao, 2017), it is likely more prevalent among adolescents with high psychosocial flourishing.

In this way, the positive experiences adolescents gain from their parents may help them develop psychological strengths that, in turn, support their charitable intentions. Synthesizing the above, we hypothesize:

**Hypothesis 2.**  *Adolescents’ psychosocial flourishing mediates the intergenerational association between parents’ psychosocial flourishing and adolescents’ CG intention.*

### 1.3. The Moderating Role of Authenticity

The cross-generational associations described above are unlikely to operate uniformly across adolescents; specific individual traits may serve as boundary conditions that strengthen or weaken them. We posited that the association between adolescents’ psychosocial flourishing and CG intention, as well as the indirect association between parental psychosocial flourishing and CG intention through adolescents’ psychosocial flourishing, depends on adolescents’ authenticity—a personality trait defined as the alignment of one’s words and actions with one’s inner thoughts and goals (Kernis & Goldman, 2006; Q. Liu et al., 2009).

Authenticity refers to being one’s “true self” (Q. Liu et al., 2009)—aligning words and actions with one’s thoughts and goals (Kernis & Goldman, 2006). Existing research has linked authenticity to higher levels of prosocial and helping behavior (Theran, 2021), as individuals with greater authenticity tend to adhere more strongly to moral principles and act in line with virtuous standards (Lefebvre & Krettenauer, 2020; Newman et al., 2015). Individuals high in authenticity tend to know themselves better, express true thoughts smoothly (Kernis & Goldman, 2006), consider others’ needs, and engage positively in interactions (Brunell et al., 2010; Tou et al., 2015). Authenticity also correlates with various indicators of psychosocial flourishing (Wood et al., 2008). Adolescents with high authenticity who experience psychosocial flourishing may exhibit genuine feelings and lasting well-being, leading to heartfelt helping behaviors such as CG. Conversely, low authenticity is associated with self-silencing (Goldner et al., 2022) and reduced emotional expressiveness (Rathi & Lee, 2021). Prolonged inauthenticity can lead to discomfort and disharmony (Gino et al., 2010), reducing optimal mental health and, consequently, the likelihood of engaging in charitable behaviors.

According to the protective–protective model (Hollister-Wagner et al., 2001), the effect of one protective factor on outcomes may vary depending on the level of another. Two competing hypotheses emerge from this framework. The protective-enhancing hypothesis suggests that one protective factor enhances the positive function of another (Li et al., 2013), implying that the promoting effect of psychosocial flourishing on CG intention would be stronger for individuals high in authenticity. Conversely, the protective-attenuating hypothesis holds that one protective factor’s effect becomes more potent when another is lower (Li et al., 2013), suggesting that psychosocial flourishing would have greater effects on CG intention among adolescents with low authenticity. Previous research indicated that those high in authenticity engage in more ethical behaviors (Kim et al., 2017; H. Zhang et al., 2019), supporting the protective-enhancing hypothesis. Thus, among adolescents with psychosocial flourishing, those high in authenticity are more likely to translate their internal resources into charitable action. Accordingly, adolescents’ authenticity may moderate the association between their psychosocial flourishing and CG intention. Furthermore, adolescents’ authenticity may influence the indirect effect of parental psychosocial flourishing on adolescents’ CG intention through adolescents’ own psychosocial flourishing. Specifically, the mediated pathway may be more pronounced for those with high levels of authenticity, because their authentic self-expression facilitates the conversion of internal psychological resources into charitable action. We therefore hypothesize:

**Hypothesis 3.**  *Adolescents’ authenticity moderates the positive association between their psychosocial flourishing and CG intention, such that the indirect association between parents’ psychosocial flourishing and adolescents’ CG intention via adolescents’ psychosocial flourishing is stronger when adolescents’ authenticity is higher.*

### 1.4. Current Study

**Figure 1:**
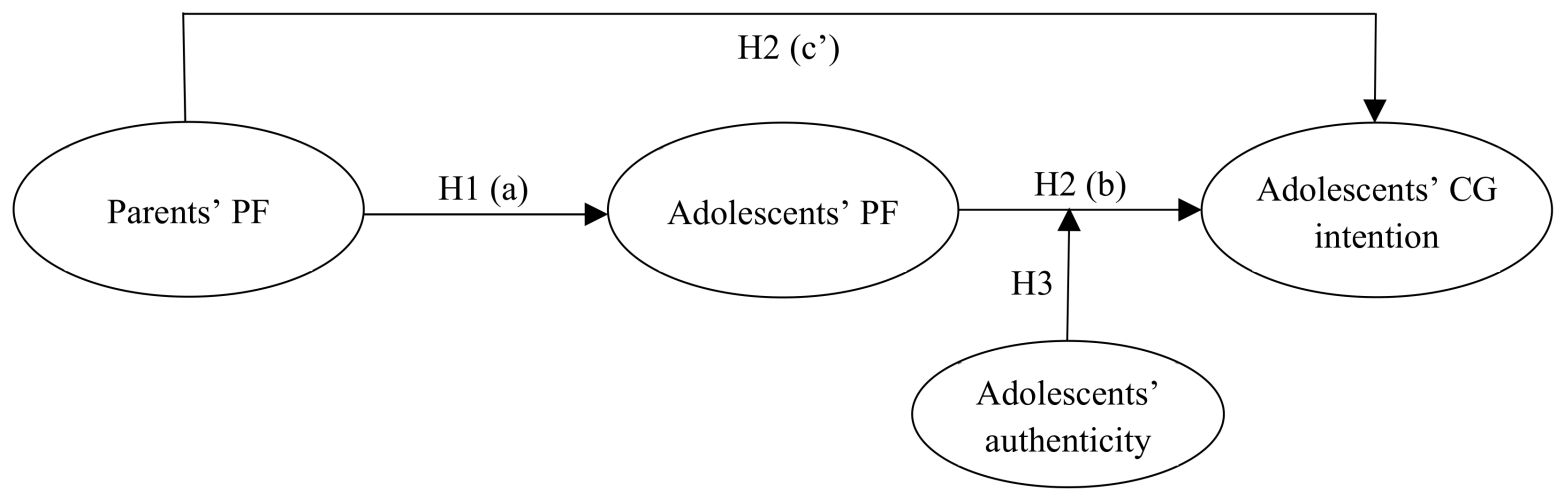
The hypothesized moderated mediation model, with paths labeled according to the hypotheses. Note. PF = psychosocial flourishing, CG = charitable giving. Path a (H1) denotes the association between parents’ PF and adolescents’ PF; paths b and c′ (H2) denote the indirect and direct associations between parents’ PF and adolescents’ CG intention; H3 denotes the moderating role of adolescents’ authenticity in the association between adolescents’ PF and CG intention.

While contemporary research has explored the factors influencing charitable behavior, the cross-generational relationship between parents’ psychosocial flourishing and adolescents’ CG intention has received limited empirical attention. Drawing on the spillover–crossover theory and an intergenerational perspective, the present research examined whether parental psychosocial flourishing is associated with adolescents’ CG intention through a moderated mediation model, as shown in Figure 1. By investigating the family-level associations through which parental psychosocial flourishing is linked to adolescents’ CG intention, this investigation contributes to the literature on adolescent charitable engagement and offers an integrative framework for understanding the patterns and boundary conditions that may characterize the cross-generational association of psychosocial flourishing and CG intention. The three hypotheses derived above were tested using the following two sub-studies. Study 1 employed a cross-sectional survey to preliminarily examine the cross-generational association of psychosocial flourishing and its link with adolescents’ CG intention, as well as the mediating role of adolescents’ psychosocial flourishing and the moderating role of their authenticity. Study 2 utilized a three-wave longitudinal design to re-examine these associations with temporal ordering and to enhance the robustness of the findings. Together, the two complementary quantitative designs balanced the strengths and limitations of each approach, thereby strengthening the overall validity of our findings.

## 2. Study 1

Study 1 aims to preliminarily examine the relationships between parental psychosocial flourishing, adolescents’ psychosocial flourishing, adolescents’ authenticity, and adolescents’ CG intention.

### 2.1. Participants and Procedure

Participants were recruited using convenience cluster sampling from two secondary vocational schools in Zhejiang Province, China, comprising 18 classes in Pinghu and 35 classes in Jiaxing. The study protocol was approved by the Academic Ethics Committee of the researchers’ university. Approximately 1300 families (2600 individuals) were initially invited. Due to practical constraints in recruiting both parents simultaneously, a single parent–adolescent dyad design was adopted, wherein one parent (either father or mother) participated alongside one adolescent per family. Informed consent was obtained from parents and adolescents before data collection; teachers were informed of the study and assisted only with scheduling and questionnaire distribution, without access to individual responses. Participants completed the online questionnaire individually, with unique dyad identifiers used solely for linking parent–adolescent data. To support complete data collection, the survey system guided participants to review any unanswered items before submission. Participants were fully informed of the research purpose and procedures, their right to withdraw at any time, and the voluntary nature of participation, with explicit assurance that declining or withdrawing would carry no academic consequences. Confidentiality assurances were given to minimize social desirability bias, and all participants received appropriate compensation for their involvement. Of the invited families, 1240 completed the questionnaires, while 60 declined participation or withdrew prior to completion. After quality control checks, 27 dyads failed two embedded attention-check items (e.g., “This question is designed to test whether you answered carefully. Please select ‘strongly agree’.” Any response other than “strongly agree” was coded as insincere responding), 18 dyads showed regular response patterns (e.g., sequential cycling through response options), 13 dyads exhibited invariant response patterns (e.g., identical answers across all items, including both positively and negatively keyed items), and 3 participants aged 20 years were excluded to ensure the sample remained strictly within the adolescent age range. After these exclusions, 1179 parent–adolescent dyads (2358 participants) provided valid data.

The adolescent samples (*M*_age_ = 16.78 years, *SD* = 0.97 years, age range = 13–19 years) comprised 685 girls and 494 boys, and the parent sample (*M*_age_ = 43.11 years, *SD* = 4.41 years, age range = 35–61 years) included 657 mothers (55.73%) and 522 fathers (44.27%). In terms of parental education levels, 53.50% of fathers and 57.20% of mothers held junior high school education or below, 31.60% of fathers and 31.20% of mothers received a high school degree, 14.00% of fathers and 11.10% of mothers had completed higher education, and 0.90% of fathers and 0.50% of mothers held a postgraduate degree. Regarding family monthly income, 25.80% of the families earned less than 5000 CNY, 46.30% earned between 5001 and 10,000 CNY, 21.50% earned between 10,001 and 20,000 CNY, and 6.40% earned more than 20,001 CNY. The sample comprised 43.20% only children and 56.80% with siblings.

### 2.2. Measures

#### 2.2.1. Psychosocial Flourishing

Psychosocial flourishing was assessed by the 8-item Flourishing Scale (Diener et al., 2010), which describes important aspects of human functioning ranging from positive relationships, to feelings of competence, to having meaning and purpose in life. Although the scale does not separately provide measures of facets of well-being, it does yield an overview of positive functioning across diverse domains that are widely believed to be important (Diener et al., 2010). An example item is “I lead a purposeful and meaningful life.” All items are rated on a 7-point Likert scale (1 = strongly disagree, 7 = strongly agree), with higher scores indicating that participants view themselves more positively in important aspects of functioning. The Cronbach’s α for children and parents were 0.95 and 0.94, respectively. In this study, the CFA index showed that the scale for parents had a satisfactory goodness of fit: *χ*^2^/*df* = 7.76, CFI = 0.98, TLI = 0.98, RMSEA = 0.076, SRMR = 0.02. Meanwhile, the CFA index showed that the scale for children also had a satisfactory goodness of fit: *χ*^2^/*df* = 7.81, CFI = 0.98, TLI = 0.97, RMSEA = 0.076, SRMR = 0.021.

#### 2.2.2. Authenticity

Authenticity was measured by the adapted 12-item Authenticity Scale (Wood et al., 2008). A representative item is “I think it is better to be yourself, than to be popular.” All items are assessed on a 7-point Likert scale (1 = strongly inconsistent, 7 = strongly consistent). Eight negatively worded items were reverse-coded, with higher scores indicating greater authenticity. Cronbach’s α was 0.79. In this study, the index of CFA showed that this scale had an acceptable goodness of fit: *χ*^2^/*df* = 6.80, CFI = 0.97, TLI = 0.95, RMSEA = 0.07, SRMR = 0.05.

#### 2.2.3. Charitable Giving Intention

Adolescents’ CG intention was assessed using three short community-based donation scenarios involving money, blood, and time. Each scenario captures a distinct type of donation, forming a multi-indicator latent construct to improve measurement breadth (H. Zhang & Zhao, 2021). A sample item reads, “With the new school year approaching, your community, in collaboration with the city’s Poverty Alleviation Education Foundation, is organizing a fundraising campaign to support children in the impoverished mountainous areas that they assist. The donations will be used to purchase school supplies for the children. If you have 200 yuan available that you don’t urgently need, would you be willing to donate?” All items are assessed on a 7-point Likert scale (1 = strongly unwilling, 7 = strongly willing). Cronbach’s α was 0.89.

### 2.3. Data Analysis

First, we tested measurement invariance between parents and adolescents, assessed common method variance, and computed descriptive statistics and bivariate correlations for all study variables using SPSS 24.0. We then performed structural equation modeling (SEM) to test the mediation model and latent moderated structural equations (LMS) to examine the moderated mediation model in Mplus 8.3. All models were estimated using maximum likelihood (ML) with full information maximum likelihood (FIML) to handle missing data (Enders & Bandalos, 2001).

To enhance the reliability of parameter estimation and adhere to methodological guidelines established by Little et al. (2002), we employed the item-to-construct balance approach to create item parcels. The specific procedure was as follows: items were first ranked in descending order based on standardized CFA factor loadings and then assigned sequentially to each parcel in a round-robin fashion, ensuring balanced representation of the latent construct across parcels. This parceling strategy mitigates model overfitting while reducing the number of parameters to be estimated, lowering sampling error, improving indicator normality, and yielding a more favorable indicator-to-sample ratio for complex models such as LMS, thereby enhancing model convergence (Little et al., 2002; Maslowsky et al., 2015). Parceling was implemented only after confirming acceptable item-level CFA fit for each scale, ensuring that no substantive local misfit was masked by aggregation.

Given that prior research has established associations between CG intention and the gender and age of both parents and adolescents, parental education level, and family monthly income (Arsyianti et al., 2017; Bekkers & Wiepking, 2011b; Wiepking & Bekkers, 2012), these variables were included as covariates in subsequent analyses to control for potential confounding effects on CG intention, thereby enabling us to isolate the independent effects of parental and adolescent psychosocial flourishing. We employed bootstrapping analysis (*N* = 5000) to estimate the significance of path coefficients, using 95% confidence intervals (CIs). Model fit was considered acceptable if the RMSEA and SRMR were below 0.08, and the CFI and TLI exceeded 0.90 (Hu & Bentler, 1999).

### 2.4. Results

#### 2.4.1. Measurement Invariance

Prior to testing the substantive models, we examined whether the psychosocial flourishing Scale assessed the same construct in parents and adolescents through multigroup confirmatory factor analysis (MGCFA) using Mplus 8.3 with MLR estimation. Configural, metric (equal factor loadings), and scalar (equal item intercepts) invariance models were estimated sequentially. Measurement invariance was considered supported when ΔCFI ≥ −0.010, ΔRMSEA ≤ 0.015, and ΔSRMR ≤ 0.030 for metric invariance, and ΔSRMR ≤ 0.010 for scalar invariance (F. F. Chen, 2007; Cheung & Rensvold, 2002). The configural model showed acceptable fit (CFI = 0.969, RMSEA = 0.054, SRMR = 0.031). The metric invariance model demonstrated adequate fit (CFI = 0.966, RMSEA = 0.053, SRMR = 0.058), with minimal changes relative to the configural model (ΔCFI = −0.003, ΔRMSEA = −0.001, ΔSRMR = 0.027). The scalar invariance model also showed acceptable fit (CFI = 0.959, RMSEA = 0.054, SRMR = 0.062), with negligible changes relative to the metric model (ΔCFI = −0.007, ΔRMSEA = 0.001, ΔSRMR = 0.004), supporting scalar invariance across parent and adolescent reports. Parents’ and adolescents’ psychosocial flourishing scores can therefore be treated as reflecting comparable latent constructs in subsequent analyses.

#### 2.4.2. Common Method Bias

Given the reliance on self-report data, common method bias was addressed through both procedural and statistical remedies. Procedurally, the study employed multi-informant reports (separate questionnaires for parents and adolescents), guaranteed anonymity and confidentiality, and incorporated reverse-scored items in the survey design to mitigate common method variance. Statistically, Harman’s single-factor test was conducted (Podsakoff et al., 2003). An unrotated exploratory factor analysis extracted five factors, with the first factor accounting for 26.71% of the total variance (well below the 40% threshold), suggesting that common method bias was not a serious concern. Furthermore, a one-factor CFA revealed poor model fit (*χ*^2^/*df* = 38.21, CFI = 0.37, TLI = 0.33, RMSEA = 0.18, SRMR = 0.21), indicating that a single latent factor does not account for the majority of the covariance among the measures. In sum, common method bias was unlikely to substantially threaten validity.

#### 2.4.3. Preliminary Analysis

As shown in Table 1, correlational analyses revealed that parents’ psychosocial flourishing was positively correlated with both adolescents’ psychosocial flourishing (*r* = 0.21, *p* < 0.001) and CG intention (*r* = 0.20, *p* < 0.001). Adolescents’ psychosocial flourishing was also positively correlated with their CG intention (*r* = 0.37, *p* < 0.001), and adolescents’ authenticity was positively related to both their psychosocial flourishing (*r* = 0.27, *p* < 0.001) and CG intention (*r* = 0.12, *p* < 0.001).

#### 2.4.4. Testing for Intergenerational Association in Psychosocial Flourishing

We first established a simple regression model with latent variables to test the effect of parents’ psychosocial flourishing on adolescents’ psychosocial flourishing. The model showed an acceptable fit to the data: *χ*^2^ = 38.88, *df* = 43, *p* = 0.65, CFI = 0.998, TLI = 0.997, RMSEA = 0.02, SRMR = 0.03. Parents’ psychosocial flourishing was positively associated with adolescents’ psychosocial flourishing (*b* = 0.23, *p* < 0.001, 95%CI [0.18, 0.29]). Hypothesis 1 was supported.

#### 2.4.5. Testing for Mediation Effect

A simple regression model with latent variables examining the direct effect of parents’ psychosocial flourishing on adolescents’ CG intention demonstrated an acceptable fit to the data: *χ*^2^ = 44.41, *df* = 43, *p* = 0.41, CFI = 0.99, TLI = 0.99, RMSEA = 0.03, SRMR = 0.03. Parents’ psychosocial flourishing was positively associated with adolescents’ CG intention (*b* = 0.23, *p* < 0.001, 95%CI [0.18, 0.28]).

**Figure 2:**
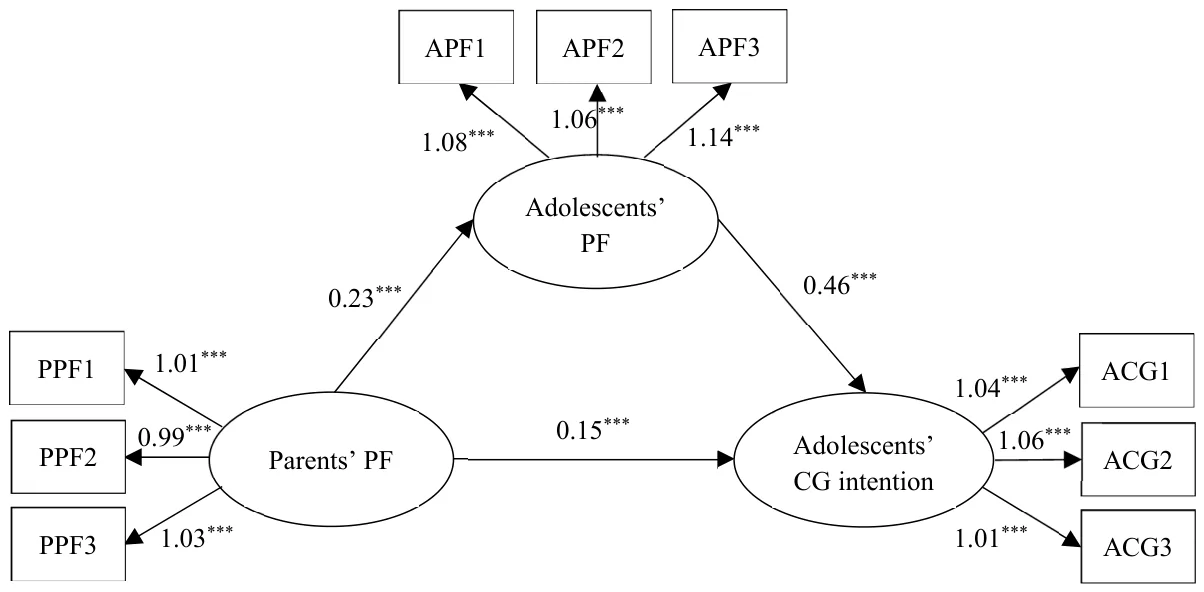
Mediation model for Study 1. Note. Unstandardized coefficients are reported; PF = parents’ psychosocial flourishing, CG = charitable giving; PPF1–PPF3 = three parcels of parents’ psychosocial flourishing; APF1–APF3 = three parcels of adolescents’ psychosocial flourishing; ACG1–ACG3 = three parcels of adolescents’ charitable giving intention. *** *p* < 0.001.

The mediation model showed a good fit to the data: *χ*^2^ = 106.43, *df* = 80, *p* = 0.03, CFI = 0.997, TLI = 0.996, RMSEA = 0.02, SRMR = 0.04. As illustrated in Figure 2, the effect of parents’ psychosocial flourishing on adolescents’ psychosocial flourishing was significant (*b* = 0.23, *p* < 0.001, 95%CI [0.18, 0.29]). When adolescents’ psychosocial flourishing was included as a mediator, it was positively associated with their CG intention (*b* = 0.46, *p* < 0.001, 95%CI [0.38, 0.53]), and the direct effect of parents’ psychosocial flourishing on adolescents’ CG intention decreased accordingly (*b* = 0.15, *p* < 0.001, 95%CI [0.09, 0.20]). Bootstrapping results are consistent with Hypothesis 2 that adolescents’ psychosocial flourishing mediated the association between parents’ psychosocial flourishing and their children’s CG intention (*b*_indirect_ = 0.11, *SE* = 0.02, *p* < 0.001, *R*^2^ = 0.24, 95%CI [0.08, 0.14]). The mediating effect accounted for 42.23% of the total effect of parents’ psychosocial flourishing on adolescents’ CG intention.

#### 2.4.6. Testing for the Moderated Mediation Model

**Figure 3:**
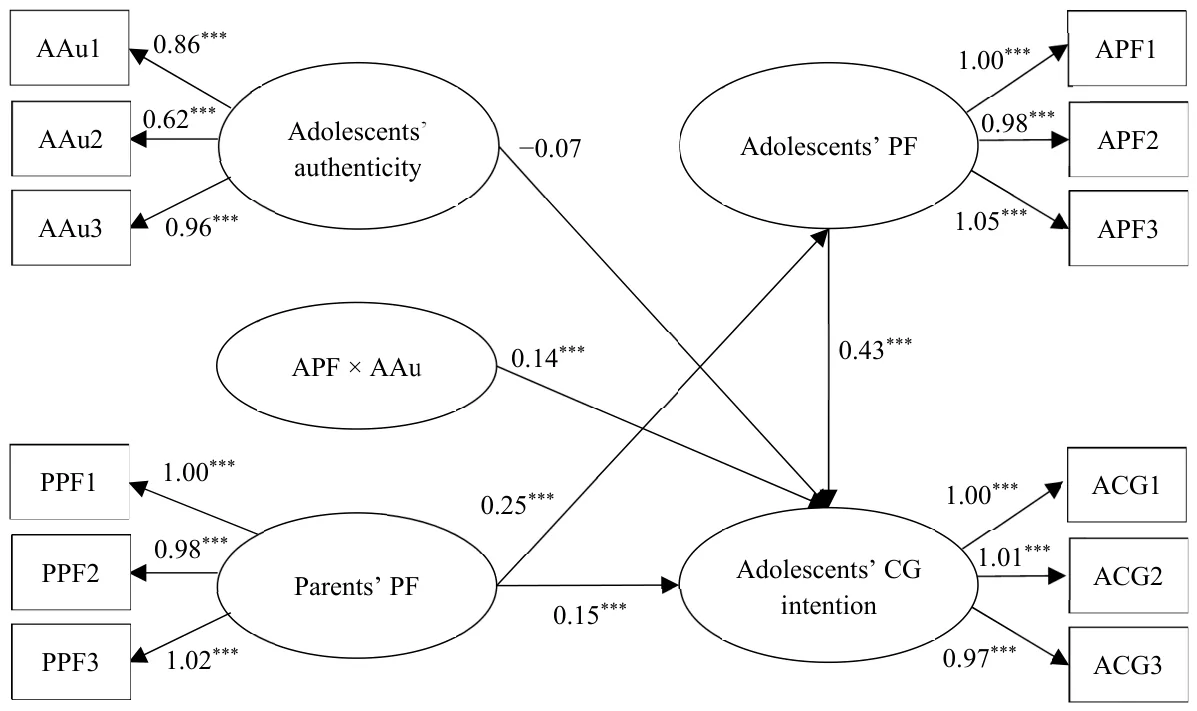
Moderated mediation model for Study 1. Note. Unstandardized coefficients are reported; PF = parents’ psychosocial flourishing, APF = adolescents’ psychosocial flourishing, AAu = adolescents’ authenticity, CG = adolescents’ charitable giving; PPF1–PPF3 = three parcels of parents’ psychosocial flourishing; APF1–APF3 = three parcels of adolescents’ psychosocial flourishing; AAu1–AAu3 = three parcels of adolescents’ authenticity; ACG1–ACG3 = three parcels of adolescents’ charitable giving intention. *** *p* < 0.001.

On the basis of the SEM measurement model tested above, we carried out LMS modeling following the procedures recommended by Maslowsky et al. (2015) to examine the moderating effect of adolescents’ authenticity. First, we tested a baseline model which excludes the latent interaction term (i.e., adolescents’ psychosocial flourishing × adolescents’ authenticity); the results showed that the model fitted the data well: *χ*^2^ = 389.90, *df* = 126, *p* < 0.001, CFI = 0.98, TLI = 0.97, RMSEA = 0.042, SRMR = 0.076. Second, we evaluated whether the model fit of the full model (i.e., the moderated mediation model including the latent interaction term) is significantly better than that of the baseline model. The value of AIC_full_ (34057.782) is less than AIC_baseline_ (34126.618). Furthermore, as indicated by the results of the log-likelihood ratio test, *χ*^2^ = −2 [(−17015.309) − (−16979.891)] = 70.836, *df* = 1, *p* < 0.001, the full model fitted the data better. Third, we used the product of coefficients approach to analyze moderated mediation effects. As illustrated in Figure 3, the interaction of adolescents’ psychosocial flourishing and their authenticity positively predicted their CG intention (*b* = 0.14, *p* < 0.001, 95%CI [0.10, 0.20]), suggesting that the positive relation between adolescents’ psychosocial flourishing and CG intention was moderated by their authenticity.

**Figure 4:**
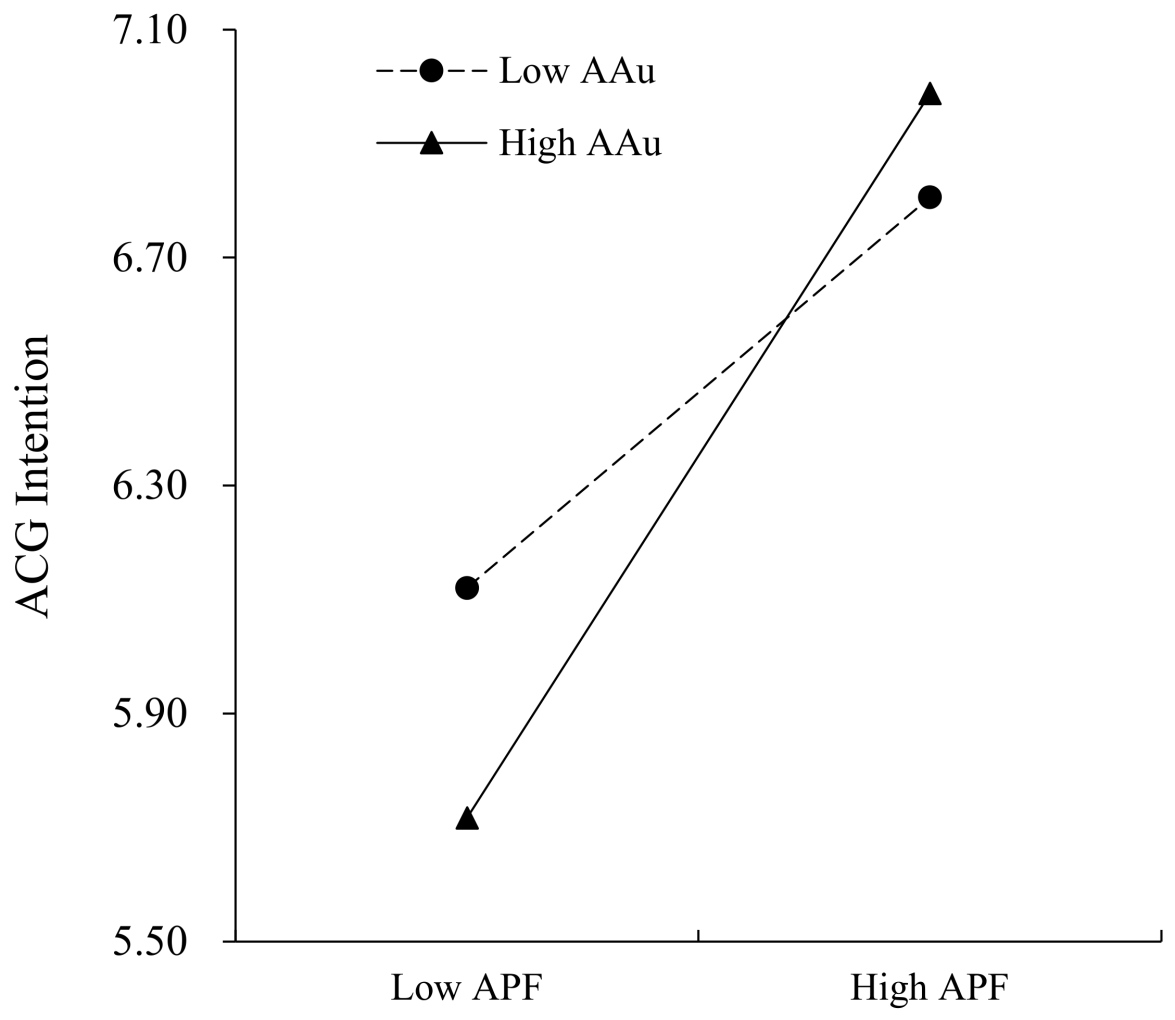
AAu moderates the relationship between APF and ACG intention. Note. Unstandardized coefficients are reported; High AAu = mean + 1 *SD*; Low AAu = mean − 1 *SD*. APF = adolescents’ psychosocial flourishing; AAu = adolescents’ authenticity; ACG = adolescents’ charitable giving.

The follow-up simple slope analysis in Figure 4 demonstrated that the positive association between adolescents’ psychosocial flourishing and CG intention was significantly stronger among adolescents with high authenticity (*b*_simple_ = 0.55, *p* < 0.001, 95% CI [0.48, 0.62]) than for adolescents with low authenticity (*b*_simple_ = 0.30, *p* < 0.001, 95% CI [0.22, 0.38]).

The bias-corrected percentile bootstrap analyses further indicated that the indirect effect of parents’ psychosocial flourishing on adolescents’ CG intention through adolescents’ psychosocial flourishing was moderated by adolescents’ authenticity (index of moderated mediation = 0.04, *SE* = 0.01, 95% CI [0.03, 0.06]). The indirect relationship between parents’ psychosocial flourishing and adolescents’ CG intention was significantly stronger for participants with high authenticity (*b* = 0.14, *SE* = 0.01, 95% CI [0.12, 0.21]), whereas this indirect relationship was weaker for participants with low authenticity (*b* = 0.07, *SE* = 0.02, 95% CI [0.05, 0.11]). This pattern of results is consistent with the moderated mediation hypothesis, as the indirect effect of parents’ psychosocial flourishing on adolescents’ CG intention through adolescents’ psychosocial flourishing was conditional on the level of adolescents’ authenticity. Hypothesis 3 was supported.

#### 2.4.7. Sensitivity Analyses: Clustering Effects

To evaluate whether classroom-level nesting biased our estimates, we computed intraclass correlation coefficients from one-way random-effects models, ICC(1), for all key variables. Two-level null models revealed small clustering effects: ICC(1) = 0.010 for parental psychosocial flourishing, 0.000 for adolescent psychosocial flourishing, 0.008 for adolescent CG intention, and 0.001 for adolescent authenticity. Corresponding design effects ranged from 1.00 to 1.21. We re-estimated all SEM and LMS models using cluster-robust standard errors (TYPE = COMPLEX in Mplus; Muthén & Muthén, 2017) with classroom as the clustering unit. The significance status of all path coefficients remained unchanged: previously significant paths stayed significant, and no non-significant paths became significant (see Supplementary Materials). Thus, accounting for clustering did not alter the substantive conclusions.

### 2.5. Discussion of Study 1

Study 1 provided preliminary support for a positive association between parental and adolescent psychosocial flourishing. It also offered preliminary evidence for a positive link between parents’ psychosocial flourishing and adolescents’ CG intention, with the mediating role of adolescents’ psychosocial flourishing and the moderating role of adolescents’ authenticity. However, the cross-sectional nature of Study 1, where all variables were measured concurrently, precluded the determination of temporal and dynamic relations among these variables. Consequently, Study 2 was conducted to further examine these patterns through a three-wave longitudinal design.

## 3. Study 2

Study 2 employed a three-wave longitudinal design to re-examine the hypothesized associations through the lens of temporal ordering, thereby strengthening the robustness of our findings in two key respects. First, while Study 1 utilized scenario-based measures to assess context-specific charitable decision-making intention, Study 2 adopted a trait-like scale to capture broader, dispositional CG intention. These conceptually aligned yet methodologically distinct operationalizations of CG intention—both rooted in the theory of planned behavior (Ajzen, 1991)—facilitate a conceptual replication of the link between parental psychosocial flourishing and adolescent CG intention across diverse methodological contexts. Second, the longitudinal design establishes temporal precedence and allows for an examination of the consistency of associations over time, offering more compelling evidence for the sequence of the hypothesized associations than the cross-sectional approach used in Study 1.

### 3.1. Participants and Procedure

Participants were recruited using convenience cluster sampling from one secondary vocational school in Lishui, Zhejiang Province, China, including 16 classes, with approval from the Academic Ethics Committee of the researcher’s university. Approximately 380 families (760 individuals) were initially invited to participate. Mirroring the procedure of Study 1, informed consent was obtained from both parents and adolescents prior to data collection, and teachers assisted with scheduling and questionnaire distribution without access to individual responses. Participants were informed of the research purpose and procedures, their right to withdraw at any time without academic consequences, and the voluntary nature of their participation. All participants completed the online questionnaire individually and confidentially. Surveys were administered at three-month intervals—a timeframe selected to balance attrition minimization with the academic calendar while reflecting the accumulation period required for enduring psychological resources (Fredrickson, 2001) and aligning with established longitudinal research on family intergenerational effects (e.g., Griffith et al., 2021). At Time 1 (T1; March 2023), a total of 356 pairs (i.e., totaling 712 participants) of adolescents and one of their parents (either parent) were enrolled and completed the survey, while 24 families declined participation or withdrew consent prior to completion. The adolescent participants (50.80% boys) were aged 16.74 (*SD* = 0.78) years on average, with an age range from 15 to 20 years. The parent sample comprised 27.50% fathers (*M*_age_ = 46.84 years, *SD* = 5.95 years) and 72.50% mothers (*M*_age_ = 43.11 years, *SD* = 4.90 years). At Time 2 (T2; June 2023) and Time 3 (T3; September 2023), surveys were conducted following the same cluster-based procedure within intact classes. Through active coordination with school administrators to schedule follow-up assessments during class time, all 356 adolescents from the T1 sample were retained across waves. However, 8 dyads were excluded from the final longitudinal data analysis due to data quality concerns observed in at least one wave: 3 dyads exhibited regular response patterns, 5 dyads showed invariant response patterns (i.e., identical answers across all items within a wave), and one additional dyad was excluded because the participant was 20 years of age, ensuring the sample remained strictly within the adolescent age range, following the same criteria as in Study 1.

The final valid longitudinal data set included 347 pairs of adolescents (49.60% girls; *M*_age_ = 16.74 years, *SD* = 0.77 years, age range: 15–19 years) and one of their parents (72.60% mothers; *M*_age_ = 43.30 years, *SD* = 4.77 years). The response rate at T1 relative to all invited families was 93.68%. In terms of parental education level, 57.60% of fathers and 59.70% of mothers held junior high school education or below, 34.0% of fathers and 31.10% of mothers received a high school degree, and the rest had a bachelor’s degree or above (8.40% of fathers and 9.30% of mothers). Regarding family monthly income, 31.70% of the families earned less than 5000 CNY, 45.80% earned between 5001 and 10,000 CNY, 15.30% earned between 10,001 and 20,000 CNY, and 7.20% earned more than 20,001 CNY. The sample comprised 21.00% only children and 79.00% with siblings.

### 3.2. Measures

#### 3.2.1. Psychosocial Flourishing

The psychosocial flourishing scale was used, as in Study 1, to evaluate both adolescents and parents’ psychosocial flourishing levels. The Cronbach’s α for parents was 0.90 at T1. The Cronbach’s α for adolescents were 0.91 at T1 and 0.97 at T2. In this study, the CFA index showed that the scale at T1 for parents had a satisfactory goodness of fit: *χ*^2^/*df* = 2.92, CFI = 0.98, TLI = 0.96, RMSEA = 0.074, SRMR = 0.031. Meanwhile, the CFA index showed that the scale at T1 for children also had a satisfactory goodness of fit: *χ*^2^/*df* = 2.96, CFI = 0.98, TLI = 0.97, RMSEA = 0.075, SRMR = 0.027.

#### 3.2.2. Authenticity

The authenticity scale was used, as in Study 1, to evaluate adolescents’ authenticity levels. Cronbach’s α was 0.88 at T2. In this study, the index of CFA showed that this scale had an acceptable goodness of fit: *χ*^2^/*df* = 3.09, CFI = 0.96, TLI = 0.94, RMSEA = 0.078, SRMR = 0.044.

#### 3.2.3. CG Intention

Adolescents’ CG intention was assessed using the adapted 5-item CG scale (Dai, 2019). A sample item is “I will take the initiative to learn about donation information.” Each item was answered on a 5-point Likert scale (1 = strongly disagree, 5 = strongly agree). Cronbach’s α was 0.83 and 0.90 at T1 and T3, respectively. In this study, the index of CFA showed that this scale at T1 had a satisfactory goodness of fit: *χ*^2^/*df* = 1.61, CFI = 0.99, TLI = 0.99, RMSEA = 0.042, SRMR = 0.01.

### 3.3. Data Analysis

Data analysis was performed using SPSS 24.0 and Mplus 8.3. Following the procedures detailed in Study 1, we assessed measurement invariance and common method variance, computed descriptive statistics and bivariate correlations, and employed SEM and LMS to test the mediation and moderated mediation models, respectively. Missing data were handled using FIML estimation (Enders & Bandalos, 2001), and all models were estimated with ML using 5000 bootstrapping resamples. In the longitudinal SEM and LMS models, adolescents’ T1 psychosocial flourishing and T1 CG intention were included as autoregressive controls to isolate change-related associations at T2 and T3. Adolescents’ gender and age, parents’ gender and age, paternal and maternal education, and family monthly income were included as covariates, with paths from all covariates to T3 CG intention freely estimated. Item parceling and model fit criteria were identical to those specified in Study 1.

### 3.4. Results

#### 3.4.1. Measurement Invariance

Prior to the main analyses, longitudinal measurement invariance was examined for the repeatedly measured constructs (adolescents’ psychosocial flourishing and CG intention) using confirmatory factor analyses across waves. Configural, metric, and scalar invariance models were compared sequentially, with invariance considered supported when ΔCFI ≥ −0.010 and ΔRMSEA ≤ 0.015, and ΔSRMR ≤ 0.030 for metric invariance and ΔSRMR ≤ 0.010 for scalar invariance (F. F. Chen, 2007; Cheung & Rensvold, 2002). For adolescents’ psychosocial flourishing, the configural model showed adequate fit (CFI = 0.946, RMSEA = 0.066, SRMR = 0.036). Constraining factor loadings across waves resulted in minimal fit deterioration (ΔCFI = −0.007, ΔRMSEA = 0.001, ΔSRMR = 0.019), and further constraining item intercepts also produced negligible changes (ΔCFI = −0.008, ΔRMSEA = 0.003, ΔSRMR = 0.007). For adolescents’ CG intention, the configural model showed adequate fit (CFI = 0.933, RMSEA = 0.078, SRMR = 0.043). Constraining factor loadings across waves resulted in negligible fit changes (ΔCFI = 0.001, ΔRMSEA = −0.005, ΔSRMR = 0.003). Partial scalar invariance was established by constraining three item intercepts to equality while freeing the intercepts of two items (items 1 and 3); the model showed acceptable fit deterioration relative to the metric model (ΔCFI = −0.007, ΔRMSEA = 0.002, ΔSRMR = 0.007). Consequently, full scalar invariance was established for adolescents’ psychosocial flourishing and partial scalar invariance for CG intention (see Supplementary Materials).

Cross-reporter invariance of the psychosocial flourishing scale between parents and adolescents at T1 was also supported. The configural model showed acceptable fit (CFI = 0.952, RMSEA = 0.070, SRMR = 0.036). The metric invariance model demonstrated adequate fit (CFI = 0.949, RMSEA = 0.068, SRMR = 0.049), with minimal changes relative to the configural model (ΔCFI = −0.003, ΔRMSEA = −0.002, ΔSRMR = 0.013). The scalar invariance model also showed acceptable fit (CFI = 0.944, RMSEA = 0.067, SRMR = 0.053), with negligible changes relative to the metric model (ΔCFI = −0.005, ΔRMSEA = −0.001, ΔSRMR = 0.004), supporting scalar invariance across parent and adolescent reports. Thus, the scale yields comparable measurements across reporters.

#### 3.4.2. Common Method Bias

Consistent with Study 1, we employed procedural remedies to minimize common method variance, including multi-informant reports (separate questionnaires for parents and adolescents), guaranteed anonymity and confidentiality, and reverse-scored items. Statistically, Harman’s single-factor test revealed seven factors with eigenvalues greater than 1, with the first factor accounting for only 25.44% of the variance. Additionally, a one-factor CFA yielded a poor model fit (*χ*^2^/*df* = 6.18, CFI = 0.40, TLI = 0.38, RMSEA = 0.122, SRMR = 0.172). These results suggest that common method bias was unlikely to pose a serious threat to the validity of our findings.

#### 3.4.3. Preliminary Analysis

As demonstrated in Table 2, correlational analyses revealed that parents’ psychosocial flourishing at T1 was positively correlated with both adolescents’ psychosocial flourishing at T1 (*r* = 0.20, *p* < 0.001)/T2 (*r* = 0.22, *p* < 0.001) and CG intention at T1 (*r* = 0.22, *p* < 0.001)/T3 (*r* = 0.24, *p* < 0.001). Adolescents’ psychosocial flourishing at T1 was also positively correlated with their CG intention at T1 (*r* = 0.38, *p* < 0.001) and at T3 (*r* = 0.22, *p* < 0.001), and adolescents’ psychosocial flourishing at T2 was also positively correlated with their CG intention at T3 (*r* = 0.31, *p* < 0.001).

#### 3.4.4. Testing for Longitudinal Intergenerational Association in Psychosocial Flourishing

As in Study 1, the simple regression model with latent variables showed an acceptable fit to the data: *χ*^2^ = 80.48, *df* = 48, *p* = 0.002, CFI = 0.99, TLI = 0.98, RMSEA = 0.044, SRMR = 0.06. After controlling for adolescents’ gender, age, parents’ gender, age, father’s education, mother’s education, family monthly income, and adolescents’ psychosocial flourishing at T1, parents’ psychosocial flourishing at T1 was positively associated with subsequent adolescents’ psychosocial flourishing at T2 (*b* = 0.19, *p* = 0.002, 95%CI [0.09, 0.29]). Hypothesis 1 was supported.

#### 3.4.5. Testing for Longitudinal Mediation Effect

We first proposed a simple regression model with latent variables to test the direct effect of parents’ psychosocial flourishing at T1 on adolescents’ CG intention at T3. The model showed an acceptable fit to the data: *χ*^2^ = 70.01, *df* = 48, *p* = 0.02, CFI = 0.99, TLI = 0.98, RMSEA = 0.036, SRMR = 0.061. Parents’ psychosocial flourishing at T1 was positively related to adolescents’ CG intention at T3 (*b* = 0.21, *p* = 0.001, 95%CI [0.10, 0.32]).

**Figure 5:**
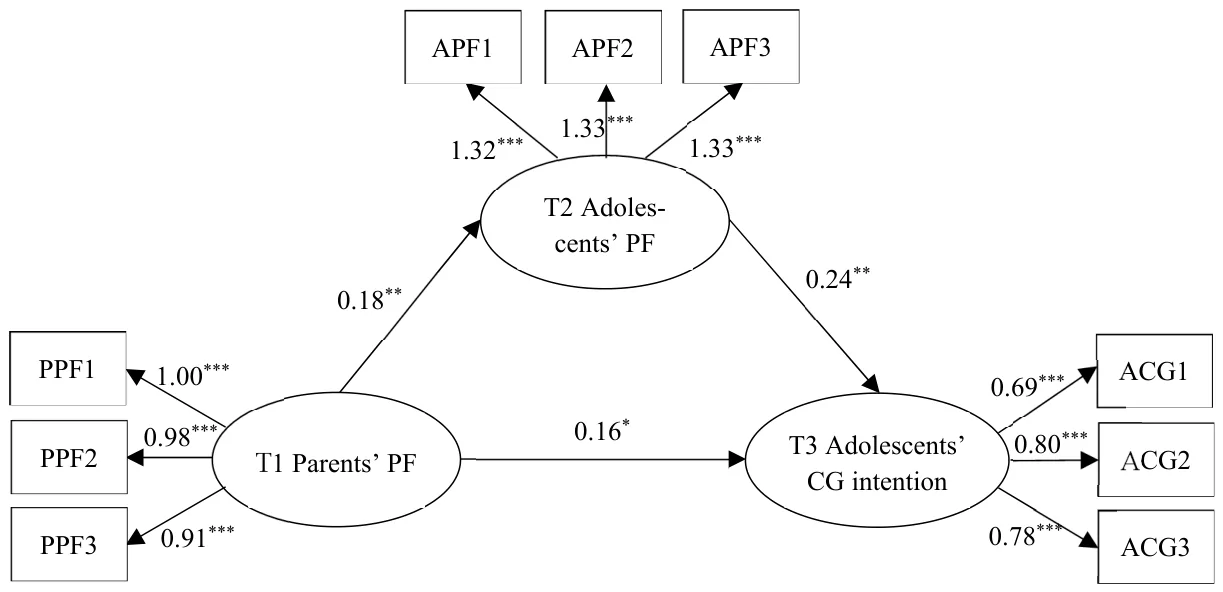
Mediation model for Study 2. Note. Unstandardized coefficients are reported; PF = parents’ psychosocial flourishing, CG = charitable giving; PPF1–PPF3 = three parcels of parents’ psychosocial flourishing; APF1–APF3 = three parcels of adolescents’ psychosocial flourishing; ACG1–ACG3 = three parcels of adolescents’ charitable giving intention. * *p* < 0.05; ** *p* < 0.01; *** *p* < 0.001.

The mediation model displayed a good fit to the data: *χ*^2^ = 135.45, *df* = 96, *p* = 0.01, CFI = 0.99, TLI = 0.98, RMSEA = 0.034, SRMR = 0.066. As illustrated in Figure 5, the effect of parents’ psychosocial flourishing at T1 on adolescents’ psychosocial flourishing at T2 was significant (*b* = 0.18, *p* = 0.001, 95%CI [0.08, 0.27]). When adolescents’ psychosocial flourishing at T2 was included as a mediator, it was positively associated with their CG intention at T3 (*b* = 0.24, *p* = 0.001, 95%CI [0.12, 0.34]), and the direct effect of parents’ psychosocial flourishing at T1 on adolescents’ CG intention at T3 decreased accordingly (*b* = 0.16, *p* = 0.02, 95%CI [0.05, 0.28]). Bootstrapping results are again consistent with Hypothesis 2 that adolescents’ psychosocial flourishing at T2 mediated the longitudinal association between parents’ psychosocial flourishing at T1 and adolescents’ CG intention at T3 (*b*_indirect_ = 0.04, *SE* = 0.02, *p* = 0.026, *R*^2^ = 0.24, 95%CI [0.02, 0.08]). The mediating effect accounted for 20.39% of the total effect of parents’ psychosocial flourishing at T1 on adolescents’ CG intention at T3.

#### 3.4.6. Testing for Longitudinal Moderated Mediation

**Figure 6:**
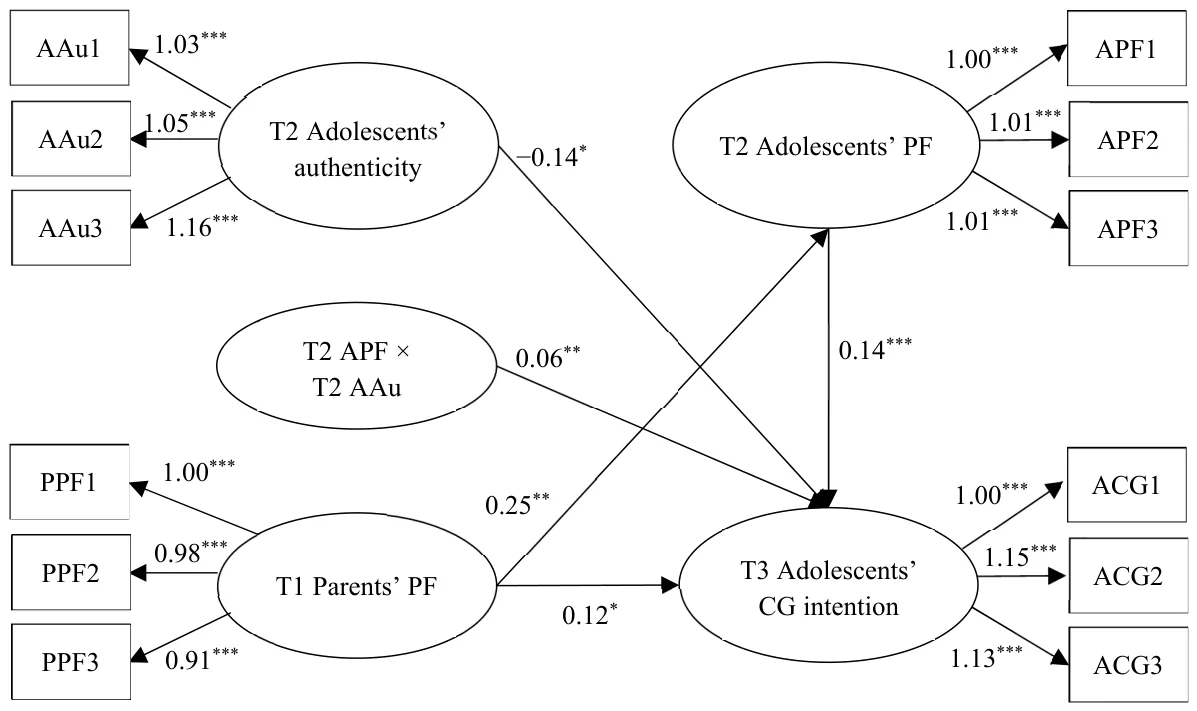
Moderated mediation model for Study 2. Note. Unstandardized coefficients are reported; PF = parents’ psychosocial flourishing, APF = adolescents’ psychosocial flourishing, AAu = adolescents’ authenticity, CG = adolescents’ charitable giving; PPF1–PPF3 = three parcels of parents’ psychosocial flourishing; APF1–APF3 = three parcels of adolescents’ psychosocial flourishing; AAu1–AAu3 = three parcels of adolescents’ authenticity; ACG1–ACG3 = three parcels of adolescents’ charitable giving intention. * *p* < 0.05; ** *p* < 0.01; *** *p* < 0.001.

The LMS analysis was conducted to examine the moderating effect of adolescents’ authenticity at T2. We first tested a baseline model which excludes the latent interaction term (i.e., adolescents’ psychosocial flourishing at T2 × adolescents’ authenticity at T2). The results showed that the model fitted the data well: *χ*^2^ = 271.059, *df* = 147, *p* < 0.001, CFI = 0.97, TLI = 0.96, RMSEA = 0.049, SRMR = 0.068. Then, we tested whether the model fit of the full model (i.e., the moderated mediation model including the latent interaction term) is significantly better than that of the baseline model. The value of AIC_full_ (9873.380) is less than AIC_baseline_ (9879.440). Furthermore, as indicated by the results of the log-likelihood ratio test, *χ*^2^ = −2[(−4888.720) − (−4884.690)] = 8.06, *df* = 1, *p* = 0.005, the full model fitted the data better. As illustrated in Figure 6, the interaction of adolescents’ psychosocial flourishing at T2 and adolescents’ authenticity at T2 positively predicted adolescents’ CG intention at T3 (*b* = 0.06, *p* = 0.005, 95%CI [0.03, 0.11]), indicating that adolescents’ authenticity at T2 moderated the association between adolescents’ psychosocial flourishing at T2 and their CG intention at T3.

**Figure 7:**
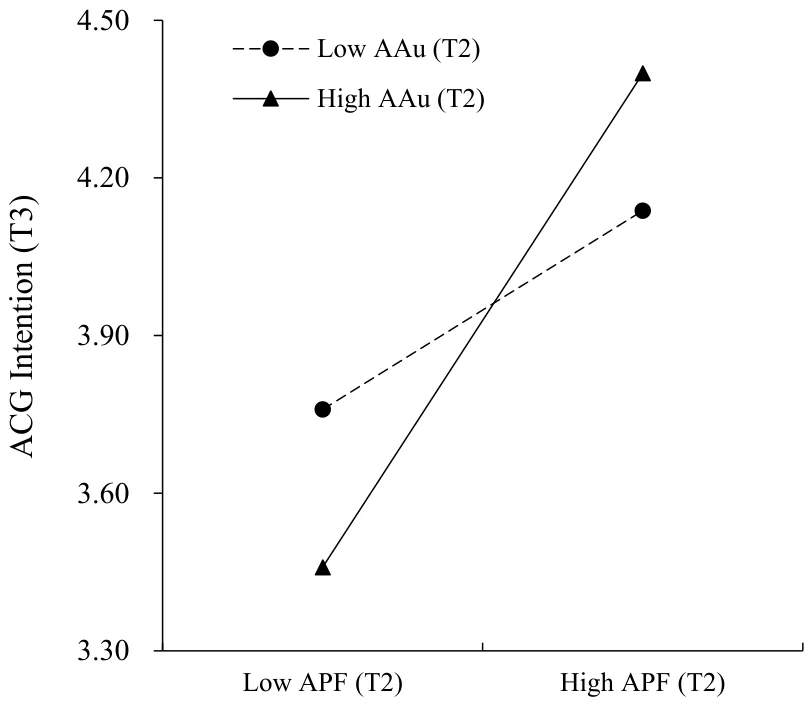
AAu at T2 moderates the link between APF at T2 and ACG intention at T3. Note. Unstandardized coefficients are reported; High AAu = mean + 1 *SD*; Low AAu = mean − 1 *SD*. APF = adolescents’ psychosocial flourishing; AAu = adolescents’ authenticity; ACG = adolescents’ charitable giving.

The follow-up simple slope analysis in Figure 7 depicted that the link between adolescents’ psychosocial flourishing at T2 and their CG intention at T3 was significantly stronger for adolescents with high authenticity (*b*_simple_ = 0.30, *p* < 0.001, 95% CI [0.20, 0.39]) than for adolescents with low authenticity (*b*_simple_ = 0.12, *p* = 0.01, 95% CI [0.03, 0.20]).

The bias-corrected percentile bootstrap analyses further supported that the indirect effect of parents’ psychosocial flourishing at T1 on adolescents’ CG intention at T3 through adolescents’ psychosocial flourishing at T2 was moderated by adolescents’ authenticity at T2 (index of moderated mediation = 0.02, *SE* = 0.01, 95% CI [0.01, 0.03]). The indirect relationship between parents’ psychosocial flourishing at T1 and adolescents’ CG intention at T3 was significantly stronger for participants with high authenticity at T2 (*b* = 0.05, *SE* = 0.02, 95% CI [0.02, 0.09]), whereas this indirect relationship was weaker for participants with low authenticity at T2 (*b* = 0.02, *SE* = 0.01, 95% CI [0.002, 0.04]). This pattern of results is consistent with the moderated mediation hypothesis, as the indirect effect of parents’ psychosocial flourishing at T1 on adolescents’ CG intention at T3 through adolescents’ psychosocial flourishing at T2 was conditional on the level of adolescents’ authenticity at T2. Hypothesis 3 was supported.

#### 3.4.7. Sensitivity Analyses: Clustering Effects

Parallel to Study 1, we examined whether classroom-level nesting biased the longitudinal estimates. Two-level null models yielded negligible ICC(1) values for the key variables: 0.002 for T1 parental psychosocial flourishing, 0.006 for T2 adolescent psychosocial flourishing, 0.005 for T3 adolescent CG intention, and 0.011 for T2 adolescent authenticity. Corresponding design effects ranged from 1.04 to 1.23. Re-estimating all longitudinal SEM and LMS models with cluster-robust standard errors (TYPE = COMPLEX in Mplus; Muthén & Muthén, 2017) did not change the significance status of any path coefficient (see Supplementary Materials). Thus, classroom clustering did not threaten the validity of the longitudinal inferences.

### 3.5. Discussion of Study 2

With the use of a three-wave longitudinal design, Study 2 replicated and extended the model in Study 1 and provided longitudinal evidence for the temporal ordering of the associations among parents’ psychosocial flourishing, adolescents’ psychosocial flourishing, CG intention, and authenticity. Parents’ psychosocial flourishing at T1 was positively associated with subsequent changes in adolescents’ psychosocial flourishing at T2, controlling for adolescents’ psychosocial flourishing at T1. Adolescents’ psychosocial flourishing at T2 mediated the longitudinal association between parents’ psychosocial flourishing at T1 and adolescents’ CG intention at T3. Furthermore, this mediation effect was more pronounced among adolescents with high authenticity. Taken together, our three hypotheses were supported both concurrently and longitudinally.

## 4. General Discussion

This study examined the intergenerational association of psychosocial flourishing between parents and adolescents and its link to adolescents’ CG intention. Drawing on the spillover–crossover theory (Bakker & Demerouti, 2018) and the broaden-and-build theory (Fredrickson, 2001), we tested a moderated mediation model in which adolescent psychosocial flourishing mediates the relationship between parents’ psychosocial flourishing and adolescents’ CG intention, and adolescent authenticity moderates this mediating pathway. Across cross-sectional (Study 1) and longitudinal (Study 2) analyses, findings consistently supported the proposed model: parents’ psychosocial flourishing was positively associated with adolescents’ psychosocial flourishing, which in turn was linked to stronger CG intention, with this indirect effect being more pronounced among adolescents higher in authenticity. These results advance the intergenerational well-being literature by exploring how parental psychosocial flourishing is related to CG intention in adolescents. Given that adolescence represents a critical window for prosocial development (Carlo & Padilla-Walker, 2020), nurturing charitable engagement during this period is essential for fostering sustainable philanthropy.

### 4.1. Intergenerational Association in Psychosocial Flourishing

In support of Hypothesis 1, the results revealed a positive statistical association between parental psychosocial flourishing and adolescent psychosocial flourishing. This finding echoes prior evidence that parental psychological and emotional profiles show consistent correlational linkage with offspring well-being (Zinovyeva, 2017). The present study offers longitudinal empirical documentation of this intergenerational associational similarity in psychosocial flourishing, contributing to the growing literature on intergenerational factors in flourishing.

The spillover–crossover theory (Bakker & Demerouti, 2018) provides a conceptual lens for interpreting this parent–adolescent association as a potential “crossover” pattern, whereby parents’ positive psychological experiences show statistical covariation with their adolescents’ well-being within the context of sustained family interactions. Theoretically, parents with higher psychosocial flourishing may exhibit greater emotional stability and warmth, and may foster a supportive family environment conducive to adolescents’ positive development; conversely, parents with lower psychosocial flourishing may display frequent negative emotional reactivity, which could contribute to a tense family atmosphere and, correspondingly, may reduce adolescents’ psychosocial flourishing levels. This pattern extends beyond transient emotional contagion; rather, parental psychological functioning shows statistical covariation with adolescents’ developmental experiences in the context of sustained parent–child interactions, shared emotional climates, and daily family routines. It is noteworthy that the key contextual factors posited to underlie these patterns, including family emotional climate and daily family routines, were not measured in the present study. These interpretations remain speculative, and future research should incorporate observational or intensive longitudinal measures of family processes to test these hypothesized mechanisms.

Given that adolescence represents a critical developmental window for psychosocial flourishing formation and enduring psychosocial adjustment (O’Connor et al., 2017; Palmer et al., 2023), the family environment plays a particularly salient role in these observed associations. The broaden-and-build theory (Fredrickson, 2001) further suggests how this potential “crossover” pattern may co-occur with stable flourishing. Although the present study did not assess parental growth-supporting behaviors or family environmental inputs, existing literature supports a tentative proposition that high psychosocial flourishing parents may coincide with adaptive environmental resources—such as autonomy support, personal growth opportunities, and adaptive behavioral modeling—that are linked to broader adolescents’ cognitive and attentional scope during family interactions. In the context of these unmeasured contextual inputs, adolescents showing higher psychosocial flourishing also displayed more enduring personal resources and adaptive cognitive and behavioral patterns related to purpose, self-acceptance, and social contribution (Eisenberg et al., 1998; Kalbfleisch, 2024). These resources include positive affect, adaptive coping capacities, and relational competencies, all of which are associated with adolescents’ psychosocial flourishing. Taken together, the spillover-crossover theory provides a conceptual heuristic for interpreting the observed intergenerational covariation, while the broaden-and-build theory offers a complementary account of how positive experiences are theoretically linked to lasting personal strengths.

### 4.2. Psychosocial Flourishing and CG Intention

Consistent with Hypothesis 2, the results revealed a direct cross-generational association between parental psychosocial flourishing and adolescent CG intention. This direct path suggests that parental well-being is associated with the family emotional climate, which in turn covaries with adolescents’ prosocial dispositions (Erreygers et al., 2019; Huta, 2012). Families of parents with higher psychosocial flourishing levels commonly feature environments marked by warmth, trust, and prosocial norms; such distal socialization contexts may coincide with adolescents’ charitable engagement separate from adolescents’ own flourishing levels. This pattern extends the spillover–crossover literature—predominantly focused on the crossover of negative states such as burnout and distress (Bakker et al., 2009; Salamon et al., 2011)—by suggesting that positive psychological resources may likewise show interpersonal covariation and be linked to prosocial behavioral intentions (Lawson et al., 2014).

Beyond this direct association, an indirect pathway operated through adolescent psychosocial flourishing. The psychosocial resources that adolescents reported in the context of their parents’ flourishing were associated with charitable behavioral intentions. This mediation pattern suggests that adolescent psychosocial flourishing emerges as a statistical factor accounting for part of the covariation between family-level well-being and individual-level CG intention. Adolescents who reported higher levels of psychosocial flourishing—encompassing not only positive affect but also self-acceptance, purpose, and perceived social contribution—also reported greater recognition of societal needs and stronger intentions to act upon charitable opportunities. This finding aligns with prior evidence documenting associations between parental and youth positive attributes and adaptive psychological and behavioral development in youth (Augustijn, 2022; Bekkers, 2007; Brown et al., 2015; Wilhelm et al., 2008).

Notably, the association between psychosocial flourishing and CG intention appears to extend beyond the purely hedonic “feel-good, do-good” pathway (Rosenhan et al., 1981). Because psychosocial flourishing integrates eudaimonic dimensions—purpose in life, autonomy, and social contribution (Keyes, 2002; Ryff, 1989)—its association with CG likely reflects more than transient positive mood. Adolescents with higher psychosocial flourishing reported greater life meaning and perceived social responsibility, as well as a broader motivational scope beyond immediate self-interest and stable prosocial values (Kakulte & Shaikh, 2023; Moradi et al., 2018). In this sense, CG represents not merely as an emotional outlet but as a behavioral expression of integrated functioning and self-realization. The cognitive and motivational resources associated with flourishing—rather than fleeting positive states alone—were concurrently linked to stronger CG intentions.

### 4.3. The Moderating Role of Authenticity

Consistent with Hypothesis 3, authenticity moderated both the direct association between adolescent psychosocial flourishing and CG intention and the indirect pathway linking parental psychosocial flourishing to adolescent CG intention. Echoing the protective-enhancing hypothesis (Li et al., 2013), the association between psychosocial flourishing and CG intention was significantly stronger among adolescents higher in authenticity.

This moderating effect reflects the resource mobilization function of authenticity. Defined as congruence between inner values and external behaviors (Kernis & Goldman, 2006; Wood et al., 2008), authenticity was associated with a stronger alignment between psychological resources and prosocial pursuits. Highly authentic adolescents possess clear self-perception, express genuine thoughts, attend to others’ needs, and maintain positive interpersonal functioning (Brunell et al., 2010; Theran, 2021). When reporting elevated psychosocial flourishing, they also reported stronger intentions for prosocial actions such as CG, which was associated with their internal resources—positive affect, purpose in life, autonomy, and social contribution. In this sense, authenticity appeared to co-occur with a stronger statistical association between internal flourishing and behavioral intentions. By contrast, adolescents with low authenticity tend to experience self-confusion and low agency, a pattern in which the positive association between psychosocial flourishing and CG intention appears attenuated. This pattern aligns with the protective-enhancing hypothesis, suggesting that authenticity is associated with greater adaptive benefits of psychosocial flourishing.

Furthermore, high authenticity coincides with a stronger mediating effect of adolescent psychosocial flourishing. Within the present theoretical model, parental psychosocial flourishing is statistically associated with adolescent psychological resources (framed as a “crossover” pattern); authenticity then moderates the subsequent statistical association between these resources and charitable intentions (framed as a “spillover” pattern). This suggests that authenticity may function as a boundary condition that is associated with whether the statistical linkage between family-level well-being resources and CG engagement is observed. While self-determination theory highlights that supportive family contexts characterized by autonomy and authenticity are linked to satisfaction of basic psychological needs and well-being (Soenens et al., 2007), the present findings suggest that adolescents’ own authentic functioning—regardless of its developmental origins—is associated with a stronger link between flourishing resources and prosocial behavioral intentions. Accordingly, authenticity not only moderates the direct association between psychosocial flourishing and CG intention but also co-occurs with a stronger developmental linkage from family psychosocial flourishing to adolescent charitable engagement.

### 4.4. Limitations and Prospects

Several limitations of this study warrant consideration and provide directions for future research. First, both studies employed convenience cluster sampling in Zhejiang Province, China, with parent–adolescent dyads nested within classrooms. Although supplementary cluster-robust analyses indicated that accounting for clustering did not alter the overall pattern of results, the inherent design effects of this sampling approach, coupled with the restricted geographic scope, may limit the external validity of the findings. Moreover, the studies were conducted within China’s collectivist cultural context, wherein family dynamics show a particularly pronounced association with adolescent behavior. This cultural specificity complicates generalizability to Western individualistic societies, where a stronger emphasis on personal autonomy may correspond with weaker family interdependence effects. Future research should therefore adopt probability sampling and more rigorous multilevel designs across diverse geographic and cultural contexts (including individualistic settings) to enhance sample representativeness and cross-cultural generalizability.

Second, the single parent–child dyad design not only precludes comprehensive family-level inferences but also constrains the model to unidirectional parent-to-adolescent pathways. Because only one parent per family participated at a single time point, we could not determine whether the observed effects were driven by maternal psychosocial flourishing, paternal psychosocial flourishing, or their interaction. In Study 2, participating parents were predominantly mothers, and the meaning of “parental psychosocial flourishing” may differ depending on which parent participated. Moreover, this design cannot capture dynamic bidirectional intergenerational processes. Future research should adopt whole-family designs with repeated assessments of both parents and employ cross-lagged panel models or random intercept cross-lagged panel models to disentangle mother-specific effects, father-specific effects, and dyadic interaction effects, as well as to test reciprocal and competitive pathways.

Third, the relatively brief longitudinal interval limits inferences about developmental trajectories and potential sleeper effects. The three-month gap, while necessary to minimize attrition, captures only short-term fluctuations in psychosocial flourishing and CG intention; it cannot address whether the observed intergenerational effects strengthen, decay, or transform over longer periods. Future research should employ follow-up designs spanning 6–12 months or longer to examine the stability of intergenerational associations and to identify sleeper effects or reciprocal influences that may emerge only across prolonged timeframes.

Fourth, although our longitudinal design establishes temporal precedence, it does not permit causal inferences. The mediation and moderated mediation patterns we observed are essentially correlational. Several unmeasured factors could plausibly account for the observed associations. Genetic relatedness between parents and adolescents may explain covariation in flourishing; shared family environments (including parenting practices, family routines, and socioeconomic conditions) and family-wide factors (such as prosocial norms and relationship quality) may simultaneously influence parental flourishing and adolescent development; selection bias may mean that families with certain characteristics are more likely to exhibit both high flourishing and high charitable intentions; and unmeasured time-varying confounders (e.g., major life events, changes in family structure, peer influences) may also concurrently affect flourishing and CG intentions. We use terms such as “intergenerational associations” and “statistical mediation” rather than “transmission” or “causal mediation” to reflect this limitation.

Fifth, the conceptual language of “spillover” and “crossover” should not be equated with empirically verified processes. They are theoretical tools that provide useful heuristic frameworks for interpreting patterns of covariation. However, the actual mechanisms linking parental flourishing to adolescent flourishing—whether through emotional contagion, social learning, shared activities, genetic similarity, or other pathways—remain untested in our design. Future research should adopt intensive longitudinal designs (e.g., daily diary methods, experience sampling methods) and genetically informed designs (e.g., twin studies, adoption studies) to disentangle these mechanisms and examine spillover–crossover processes more directly.

Finally, the reliance on self-reported measures introduces an intention–behavior gap in assessing adolescents’ charitable engagement. Our study assessed CG intention rather than actual CG behaviors. Although behavioral intention is widely recognized as a robust predictor of actual behavior (Ajzen, 1991), certain factors may exhibit stronger predictive validity for actual behaviors than for self-reported intentions (Kish-Gephart et al., 2010). Thus, future research should bridge this intention–behavior gap by developing and validating methodologies that directly capture actual charitable engagement, thereby enhancing the substantive validity of research conclusions.

### 4.5. Implications

Despite limitations, the present study carries implications at both theoretical and practical levels. Theoretically, first, it highlights a previously underexplored intergenerational pathway associated with parental well-being and adolescent charitable intention. The observed pattern indicates that parental psychosocial flourishing is correlated with adolescent CG intention, both directly and indirectly through adolescent psychosocial flourishing, which may help advance prosocial development theories beyond unidirectional socialization models toward dynamic, resource-based frameworks. Second, by replicating this pathway within a large Chinese sample, the study extends the flourishing literature beyond its predominantly Western base (Augustijn, 2022; Moradi et al., 2018; Zinovyeva, 2017). The cross-cultural generalizability of psychosocial flourishing intergenerational association and its prosocial consequences warrants continued attention, particularly given cultural variations in family interdependence and charitable norms. Third, the study integrates two complementary theoretical perspectives: the spillover–crossover theory, which describes how psychological experiences may covary between family members, and the broaden-and-build theory, which portrays how these experiences are theoretically linked to lasting psychological strengths and charitable behavioral intentions. This pairing offers a useful starting point for future research to explore how family-level psychosocial flourishing may be associated with cross-generational behavioral tendencies.

Practically, the associative patterns observed in this study may provide preliminary cues for future intervention research. The positive association between parental psychosocial flourishing and adolescents’ CG intention suggests that parental psychosocial flourishing warrants attention in future interventions. Likewise, the mediating role of adolescent psychosocial flourishing indicates that this variable merits consideration in intervention design. The moderating effect of authenticity further points to this trait as a potentially meaningful boundary condition for future work. Although personality is generally considered relatively stable, prior research has demonstrated its plasticity (Kornadt et al., 2019). Thus, authenticity and its association with CG intention may constitute a promising avenue for future intervention research, though whether interventions targeting these variables would enhance adolescents’ CG intention remains to be empirically tested.

## 5. Conclusions

This study systematically examined the intergenerational association between parental psychosocial flourishing and adolescents’ CG intention by integrating cross-sectional and three-wave longitudinal designs. Results suggested a significant positive intergenerational link between parental and adolescent psychosocial flourishing. Adolescents’ psychosocial flourishing statistically mediated the association between parental psychosocial flourishing and adolescents’ CG intention, and adolescents’ authenticity positively moderated this indirect effect, with the intergenerational indirect association being more pronounced among adolescents with higher authenticity. These findings enrich understanding of how flourishing resources within the family relate to adolescent charitable engagement from an intergenerational perspective and expand the application of the spillover–crossover framework to patterns involving positive psychological resources. Future research would benefit from more rigorous long-term longitudinal monitoring and systematic investigation. Interventions focused on psychosocial flourishing and adolescent authenticity may shed additional light on the observed patterns and inform efforts regarding adolescent charitable engagement.

## Figures and Tables

**Table 1:** Descriptive statistics and correlations among study variables.

Variables	1	2	3	4	5	6	7	8	9
1. Age of adolescents	—								
2. Age of parents	0.13 ***	—							
3. Father’s education	0.07 **	−0.08 **	—						
4. Mother’s education	0.03	−0.09 **	0.60 ***	—					
5. Family income	0.02	−0.10 ***	0.19 ***	0.15 ***	—				
6. PPF	−0.02	−0.01	0.01	0.03	0.11 ***	—			
7. APF	0.12 ***	−0.03	0.08 **	0.14 ***	0.05	0.21 ***	—		
8. AAu	−0.003	−0.03	−0.003	0.06	0.05	0.04	0.27 ***	—	
9. ACG intention	−0.04	0.03	−0.04	−0.02	0.001	0.20 ***	0.37 ***	0.12 ***	—
*M*	16.78	43.11	1.62	1.55	2.09	5.33	4.97	4.20	4.41
*SD*	0.97	4.41	0.76	0.71	0.85	1.05	1.15	0.86	1.24

Note. Education: 1 = junior high school education or below, 2 = high school degree, 3 = bachelor degree and 4 = postgraduate degree. Family income: 1 = less than 5000 CNY, 2 = 5001–10,000 CNY, 3 = 10,001–20,000 CNY, and 4 = above 20,001 CNY. PPF = parents’ psychosocial flourishing, APF = adolescents’ psychosocial flourishing, AAu = adolescents’ authenticity, ACG = adolescents’ charitable giving. ** *p* < 0.01; *** *p* < 0.001.

**Table 2:** Descriptive statistics and correlations among study variables.

Variables	1	2	3	4	5	6	7	8	9	10	11
1. Age of adolescents	—										
2. Age of parents	0.05	—									
3. Father’s education	−0.05	−0.03	—								
4. Mother’s education	−0.01	−0.19 ***	0.62 ***	—							
5. Family income	0.04	−0.11 *	0.15 **	0.20 ***	—						
6. T1 PPF	0.09	0.06	0.06	0.09	−0.06	—					
7. T1 APF	0.09	0.08	0.05	0.01	0.02	0.20 ***	—				
8. T1 ACG intention	0.09	0.09	0.14 **	0.12 *	0.01	0.22 ***	0.38 ***	—			
9. T2 APF	−0.11 *	0.10	0.04	0.03	−0.001	0.22 ***	0.45 ***	0.21 ***	—		
10. T2 AAu	0.02	0.03	0.02	0.05	0.04	0.07	0.17 **	−0.03	0.47 ***	—	
11. T3 ACG intention	−0.002	0.06	0.06	0.09	0.04	0.24 ***	0.22 ***	0.40 ***	0.31 ***	0.10	—
*M*	16.74	44.37	1.51	1.50	1.98	5.22	4.82	3.33	4.47	4.00	3.20
*SD*	0.77	5.06	0.66	0.69	0.87	1.01	1.13	0.79	1.54	1.13	0.92

Note. Education: 1 = junior high school education or below, 2 = high school degree, 3 = bachelor degree and 4 = postgraduate degree. Family income: 1 = less than 5000 CNY, 2 = 5001–10,000 CNY, 3 = 10,001–20,000 CNY, and 4 = above 20,001 CNY. PPF = parents’ psychosocial flourishing, APF = adolescents’ psychosocial flourishing, AAu = adolescents’ authenticity, ACG = adolescents’ charitable giving. * *p* < 0.05; ** *p* < 0.01; *** *p* < 0.001.

## Data Availability

The raw data supporting the conclusions of this article will be made available by the authors on request.

## Supplementary Materials

The following supporting information can be downloaded at: https://www.mdpi.com/article/10.3390/bs16091480/s1, Table S1. Item assignment for each parcel in Study 1. Table S2. Item assignment for each parcel in Study 2. Figure S1. Mediation model for Study 1. Figure S2. Moderated mediation model for Study 1. Table S3. Longitudinal Measurement Invariance Across Waves. Table S4. Cross-Reporter Measurement Invariance (Parent vs. Adolescent) at T1. Figure S3. Mediation model for Study 2. Figure S4. Moderated mediation model for Study 2.

## Abbreviations

The following abbreviations are used in this manuscript:

CG	Charitable giving
CIs	Confidence intervals
