# Supplementary material for: Parental Psychosocial Flourishing and Adolescents’ Charitable Giving Intention: The Roles of Adolescent Psychosocial Flourishing and Authenticity
Source: Behav Sci (Basel). 2026 Aug 25;16(9):1480. doi: 10.3390/bs16091480 (PMC13603507; doi:10.3390/bs16091480)
Supplement: Supplementary file 1 [file behavsci-16-01480-s001.zip › behavsci-4492402-supplementary.pdf]

Supplementary Materials

Exact item-to-parcel allocations

All parcels were created using the item-to-construct balance approach (Little et al., 2002). Specifically, items were ranked by standardized CFA factor loadings in descending order and then assigned sequentially to parcels in round-robin fashion, ensuring that each parcel contains a balanced representation of high, medium, and low loadings. The resulting allocations are:

Table S1. Item assignment for each parcel in Study 1

Construct	Parcel	Items (by original item number)	N of items
Parents' PF (PPF)	Parcel 1	Item 07, Item 08, Item 02	3
	Parcel 2	Item 03, Item 01	2
	Parcel 3	Item 06, Item 05, Item 04	3
Adolescents' PF (APF)	Parcel 1	Item 03, Item 01, Item 02	3
	Parcel 2	Item 05, Item 06, Item 08	3
	Parcel 3	Item 07, Item 04	2
Adolescents' Authenticity (AAU)	Parcel 1	Item 07, Item 08, Item 12, Item 01	4
	Parcel 2	Item 11, Item 06, Item 04, Item 03	4
	Parcel 3	Item 10, Item 09, Item 05, Item 02	4
Adolescents' CG Intention (ACG)	ACG1	Scenario 1 (monetary donation)	1
	ACG2	Scenario 2 (blood donation)	1
	ACG3	Scenario 3 (time donation)	1

Table S2. Item assignment for each parcel in Study 2

Construct	Parcel	Items (by original item number)	N of items
T1 Parents' PF (PPF)	Parcel 1	Item 05, Item 07, Item 02	3
	Parcel 2	Item 06, Item 03, Item 01	3
	Parcel 3	Item 08, Item 04	2
T2 Adolescents' PF (APF)	Parcel 1	Item 05, Item 01, Item 02	3
	Parcel 2	Item 07, Item 06, Item 08	3
	Parcel 3	Item 03, Item 04	2
T2 Adolescents' Authenticity (AAU)	Parcel 1	Item 07, Item 09, Item 08, Item 01	4
	Parcel 2	Item 06, Item 10, Item 12, Item 04	4
	Parcel 3	Item 03, Item 11, Item 05, Item 02	4
T3 Adolescents' CG Intention (ACG)	Parcel 1	Item 04, Item 05	2
	Parcel 2	Item 01, Item 03	2
	Parcel 3	Item 02	1

Study 1

Study 1 Primary Model – Mediation SEM Syntax:

```
TITLE: Mediation model (cross-sectional)
DATA:
   FILE = Charity.dat;
VARIABLE:
   NAMES = Agender Aage Pgender Page Fedu Medu Fincome
               x1-x3 m1-m3 y1-y3 z1-z3 class;
   USEVARIABLE = Agender Aage Pgender Page Fedu Medu Fincome
                        x1-x3 m1-m3 y1-y3;
   MISSING = ALL(-999);
ANALYSIS:
   BOOTSTRAP = 5000;
MODEL:
   ! Latent factor definitions
   PPF BY x1-x3*;
   APF BY m1-m3*;
   ACG BY y1-y3*;
   PPF@1; APF@1; ACG@1;
   ! Core mediation paths
   ACG ON PPF APF;
   APF ON PPF;
! Covariate controls on endogenous factors
   ACG ON Agender Aage Pgender Page Fedu Medu Fincome;
MODEL INDIRECT:
   ACG IND APF PPF;
OUTPUT:
   STANDARDIZED CINTERVAL(BCBOOTSTRAP);
```

Study 1 Primary Model – Moderated Mediation LSM Syntax:

```
TITLE: LMS full model (cross-sectional)
  DATA:
    FILE = Charity.dat;
  VARIABLE:
    NAMES = Agender Aage Pgender Page Fedu Medu Fincome
              x1-x3 m1-m3 y1-y3 z1-z3 class;
    USEVARIABLE = Agender Aage Pgender Page Fedu Medu Fincome
                    x1-x3 m1-m3 y1-y3 z1-z3;
    MISSING = ALL(-999);
  ANALYSIS:
    TYPE = RANDOM;
    ESTIMATOR = ML;
    ALGORITHM = INTEGRATION;
    BOOTSTRAP = 5000;
    PROCESSORS = 8;
    CONVERGENCE = 0.1;
  MODEL:
    ! Latent factor definitions (aligned with baseline)
    PPF   BY x1-x3;
    APF   BY m1-m3;
    AAU   BY z1-z3*;
    ACG   BY y1-y3;
    AAU@1;
    ! Latent interaction term
    MZ | APF XWITH AAU;
    ! Core paths (labeled for MODEL CONSTRAINT)
    APF   ON PPF(a1);
    ACG   ON APF(b1);
    ACG   ON AAU(b2);
    ACG   ON MZ(b3);
    ACG   ON PPF(c);
    ! Covariate controls on endogenous factors (from baseline)
    ACG   ON Agender Aage Pgender Page Fedu Medu Fincome;
  MODEL CONSTRAINT:
    NEW(LOW_AAU MED_AAU HIGH_AAU
        IND_LOWAAU IND_MEDAAU IND_HIAAU
        IMAPF
        TOT_LOWAAU TOT_MEDAAU TOT_HIAAU);
    LOW_AAU = -1;
    MED_AAU = 0;
    HIGH_AAU = 1;
    IND_LOWAAU = a1*b1 + a1*b3*LOW_AAU;
```

```
    IND_MEDAAU = a1*b1 + a1*b3*MED_AAU;
    IND_HIAAU = a1*b1 + a1*b3*HIGH_AAU;
    IMAPF = a1*b3;
    TOT_LOWAAU = IND_LOWAAU + c;
    TOT_MEDAAU = IND_MEDAAU + c;
    TOT_HIAAU = IND_HIAAU + c;
    PLOT(LOMOD MEDMOD HIMOD);
    LOOP(XVAL,-3,3,0.1);
    LOMOD = IND_LOWAAU*XVAL;
    MEDMOD = IND_MEDAAU*XVAL;
    HIMOD = IND_HIAAU*XVAL;
  PLOT:
    TYPE = PLOT2;
  OUTPUT:
    CINTERVAL(BCBOOTSTRAP);
```

OUTPUT:

MODEL RESULTS

				Two-Tailed
	Estimate	S.E.	Est./S.E.	P-Value
PPF BY				
X1	1.000	0.000	999.000	999.000
X2	0.977	0.024	40.225	0.000
X3	1.021	0.020	52.288	0.000
APF BY				
M1	1.000	0.000	999.000	999.000
M2	0.980	0.022	44.364	0.000
M3	1.050	0.023	46.271	0.000
AAU BY				
Z1	0.858	0.024	35.627	0.000
Z2	0.617	0.021	28.908	0.000
Z3	0.960	0.031	31.326	0.000
ACG BY				
Y1	1.000	0.000	999.000	999.000
Y2	1.009	0.038	26.329	0.000
Y3	0.970	0.036	26.563	0.000
APF ON				
PPF	0.251	0.038	6.616	0.000
ACG ON				
APF	0.428	0.037	11.579	0.000
AAU	-0.065	0.046	-1.406	0.160
MZ	0.144	0.034	4.285	0.000
PPF	0.151	0.027	5.511	0.000
ACG ON				

AGENDER	-0.311	0.063	-4.908	0.000
AAGE	-0.088	0.034	-2.560	0.010
PGENDER	-0.038	0.057	-0.661	0.509
PAGE	0.006	0.007	0.928	0.353
FEDU	-0.066	0.056	-1.179	0.239
MEDU	-0.063	0.060	-1.041	0.298
FINCOME	-0.023	0.045	-0.514	0.607
AAU WITH				
PPF	0.044	0.031	1.425	0.154
Intercepts				
X1	5.304	0.031	172.959	0.000
X2	5.455	0.031	176.048	0.000
X3	5.269	0.034	155.646	0.000
M1	4.895	0.034	145.067	0.000
M2	5.060	0.031	164.574	0.000
M3	4.953	0.035	139.659	0.000
Y1	6.340	0.581	10.914	0.000
Y2	6.393	0.577	11.076	0.000
Y3	6.331	0.559	11.320	0.000
Z1	4.177	0.031	133.376	0.000
Z2	4.329	0.022	198.017	0.000
Z3	4.101	0.029	140.222	0.000
Variances				
PPF	1.025	0.047	21.667	0.000
AAU	1.000	0.000	999.000	999.000
Residual Variances				
X1	0.178	0.022	8.255	0.000
X2	0.280	0.028	10.146	0.000
X3	0.192	0.023	8.398	0.000
M1	0.295	0.036	8.286	0.000
M2	0.197	0.029	6.880	0.000
M3	0.238	0.042	5.639	0.000
Y1	0.678	0.056	12.025	0.000
Y2	0.367	0.080	4.608	0.000
Y3	0.505	0.063	8.076	0.000
Z1	0.255	0.026	9.893	0.000
Z2	0.314	0.017	18.235	0.000
Z3	0.216	0.028	7.664	0.000
APF	1.171	0.055	21.228	0.000
ACG	1.062	0.067	15.789	0.000
New/Additional Parameters				
LOW_AAU	-1.000	0.000	*********	0.000
MED_AAU	0.000	0.000	0.000	1.000
HIGH_AAU	1.000	0.000	*********	0.000

IND_LOWA	0.071	0.017	4.091	0.000
IND_MEDA	0.107	0.019	5.767	0.000
IND_HIAA	0.143	0.024	6.089	0.000
IMAPF	0.036	0.009	3.975	0.000
TOT_LOWA	0.222	0.030	7.530	0.000
TOT_MEDA	0.258	0.030	8.727	0.000
TOT_HIAA	0.294	0.032	9.101	0.000

CONFIDENCE INTERVALS OF MODEL RESULTS

	Lower .5%	Lower 2.5%	Lower 5%	Estimate	Upper 5%	Upper 2.5%	Upper .5%
PPF BY							
X1	1.000	1.000	1.000	1.000	1.000	1.000	1.000
X2	0.935	0.935	0.942	0.977	1.027	1.027	1.029
X3	0.981	0.982	0.991	1.021	1.049	1.050	1.051
APF BY							
M1	1.000	1.000	1.000	1.000	1.000	1.000	1.000
M2	0.921	0.921	0.938	0.980	1.012	1.020	1.032
M3	1.005	1.005	1.005	1.050	1.075	1.083	1.098
AAU BY							
Z1	0.811	0.813	0.821	0.858	0.906	0.918	0.919
Z2	0.568	0.578	0.589	0.617	0.656	0.656	0.656
Z3	0.898	0.909	0.921	0.960	1.006	1.006	1.006
ACG BY							
Y1	1.000	1.000	1.000	1.000	1.000	1.000	1.000
Y2	0.926	0.926	0.948	1.009	1.081	1.088	1.103
Y3	0.903	0.903	0.903	0.970	1.017	1.023	1.036
APF ON							
PPF	0.187	0.200	0.205	0.251	0.318	0.348	0.348
ACG ON							
APF	0.350	0.364	0.369	0.428	0.496	0.496	0.498
AAU	-0.179	-0.158	-0.137	-0.065	0.025	0.025	0.027
MZ	0.076	0.095	0.102	0.144	0.200	0.202	0.202
PPF	0.084	0.084	0.114	0.151	0.196	0.205	0.215
ACG ON							
AGENDER	-0.423	-0.423	-0.415	-0.311	-0.204	-0.192	-0.160
AAGE	-0.155	-0.127	-0.124	-0.088	-0.018	-0.017	-0.017
PGENDER	-0.163	-0.163	-0.163	-0.038	0.021	0.029	0.058
PAGE	-0.007	-0.005	-0.002	0.006	0.018	0.018	0.025
FEDU	-0.190	-0.188	-0.165	-0.066	0.024	0.040	0.070
MEDU	-0.181	-0.181	-0.174	-0.063	0.048	0.051	0.053
FINCOME	-0.115	-0.115	-0.105	-0.023	0.037	0.050	0.069
AAU WITH							
PPF	-0.031	-0.005	0.003	0.044	0.099	0.108	0.108
Intercepts							

X1	5.231	5.231	5.231	5.304	5.343	5.346	5.366
X2	5.398	5.398	5.398	5.455	5.498	5.511	5.527
X3	5.211	5.211	5.215	5.269	5.318	5.324	5.351
M1	4.810	4.831	4.843	4.895	4.936	4.969	4.996
M2	5.005	5.014	5.015	5.060	5.120	5.127	5.127
M3	4.891	4.903	4.911	4.953	5.040	5.040	5.040
Y1	4.945	4.945	4.945	6.340	7.077	7.227	7.348
Y2	5.018	5.018	5.053	6.393	7.164	7.190	7.410
Y3	4.991	4.991	4.991	6.331	6.994	7.182	7.227
Z1	4.097	4.119	4.130	4.177	4.231	4.237	4.247
Z2	4.273	4.297	4.303	4.329	4.364	4.364	4.364
Z3	4.010	4.042	4.066	4.101	4.150	4.159	4.159
Variances							
PPF	0.913	0.916	0.967	1.025	1.091	1.105	1.108
AAU	1.000	1.000	1.000	1.000	1.000	1.000	1.000
Residual Variances							
X1	0.134	0.146	0.147	0.178	0.223	0.225	0.226
X2	0.205	0.226	0.242	0.280	0.327	0.330	0.330
X3	0.131	0.131	0.154	0.192	0.224	0.228	0.247
M1	0.234	0.234	0.244	0.295	0.346	0.346	0.395
M2	0.143	0.143	0.145	0.197	0.249	0.252	0.272
M3	0.125	0.178	0.183	0.238	0.321	0.330	0.330
Y1	0.566	0.597	0.600	0.678	0.795	0.795	0.836
Y2	0.179	0.254	0.278	0.367	0.520	0.576	0.576
Y3	0.387	0.438	0.459	0.505	0.644	0.644	0.644
Z1	0.170	0.170	0.170	0.255	0.280	0.282	0.291
Z2	0.280	0.288	0.294	0.314	0.354	0.354	0.354
Z3	0.166	0.166	0.176	0.216	0.257	0.263	0.273
APF	1.044	1.087	1.099	1.171	1.275	1.289	1.289
ACG	0.927	0.976	0.988	1.062	1.232	1.232	1.232
New/Additional Parameters							
LOW_AAU	-1.000	-1.000	-1.000	-1.000	-1.000	-1.000	-1.000
MED_AAU	0.000	0.000	0.000	0.000	0.000	0.000	0.000
HIGH_AAU	1.000	1.000	1.000	1.000	1.000	1.000	1.000
IND_LOWA	0.035	0.046	0.050	0.071	0.112	0.115	0.115
IND_MEDA	0.080	0.084	0.090	0.107	0.154	0.154	0.154
IND_HIAA	0.107	0.117	0.122	0.143	0.211	0.211	0.211
IMAPF	0.016	0.023	0.025	0.036	0.057	0.059	0.059
TOT_LOWA	0.139	0.139	0.139	0.222	0.253	0.264	0.289
TOT_MEDA	0.173	0.173	0.211	0.258	0.307	0.319	0.334
TOT_HIAA	0.206	0.245	0.257	0.294	0.350	0.368	0.368

<u>All SEM and LMS models in Study 1 were re-estimated using cluster-robust standard errors. Results of the cluster-robust sensitivity analyses are reported below:</u>

Testing for Intergenerational Association in Psychosocial Flourishing

We first established a simple regression model with latent variables to test the effect of parents' psychosocial flourishing on adolescents' psychosocial flourishing, using TYPE = COMPLEX to account for the nesting of dyads within classrooms and 5,000 bootstrap draws for bias-corrected confidence intervals. Parents' psychosocial flourishing was positively associated with adolescents' psychosocial flourishing (b = 0.23, p < 0.001, 95%CI [0.12, 0.28]). Hypothesis 1 was supported.

Testing for Mediation Effect

A simple regression model with latent variables was then estimated to examine the direct effect of parents' psychosocial flourishing on adolescents' CG intention. Results showed that parents' psychosocial flourishing was positively associated with adolescents' CG intention (b = 0.23, p < 0.001, 95%CI [0.18, 0.27]).

As illustrated in Figure S1, the effect of parents' psychosocial flourishing on adolescents' psychosocial flourishing was significant (b = 0.23, p < 0.001, 95%CI [0.18, 0.29]). When adolescents' psychosocial flourishing was included as a mediator, it was positively associated with their CG intention (b = 0.46, p < 0.001, 95%CI [0.39, 0.52]), and the direct effect of parents' psychosocial flourishing on adolescents' CG intention decreased accordingly (b = 0.15, p < 0.001, 95%CI [0.10, 0.19]). Bootstrapping results are consistent with Hypothesis 2 that adolescents' psychosocial flourishing mediated the association between parents' psychosocial flourishing and their children's CG intention ($b_{indirect}$ = 0.11, SE = 0.02, p < 0.001, R^2 = 0.25, 95%CI [0.08, 0.14]). The mediating effect accounted for 42.34% of the total effect of parents' psychosocial flourishing on adolescents' CG intention.

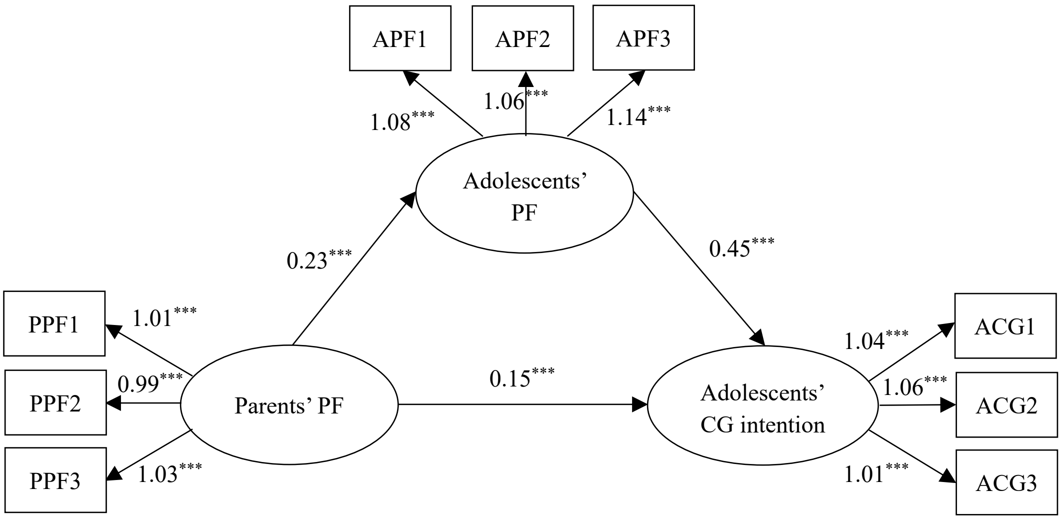

Figure S1. Mediation model for Study 1. *Note.* Unstandardized coefficients are reported; PF = parents' psychosocial flourishing, CG = charitable giving; PPF1-PPF3 = three parcels of parents' psychosocial flourishing; APF1-APF3 = three parcels of adolescents' psychosocial flourishing; ACG1-ACG3 = three parcels of adolescents' charitable giving intention. $^{***}p < 0.001$.

Testing for the Moderated Mediation Model

On the basis of the SEM measurement model tested above, we carried out LMS modeling following the procedures recommended by Maslowsky et al. (2015) to examine the moderating effect of adolescents' authenticity. First, we tested a baseline model which excludes the latent interaction term (i.e., adolescents' psychosocial flourishing × adolescents' authenticity), the results showed that the model fitted data well: χ^2 = 327.917, df = 126, p < 0.001, CFI = 0.97, TLI = 0.97, RMSEA = 0.037, SRMR = 0.076. Second, we evaluated whether

the model fit of the full model (i.e., the moderated mediation model including the latent interaction term) is significantly better than that of the baseline model. The value of AIC_{full} (34057.782) is less than $AIC_{baseline}$ (34126.618). Furthermore, as indicated by the results of the log-likelihood ratio test, $\chi^2 = -2$ [(−17015.309) − (−16979.891)] = 70.836, $df = 1$, $p < 0.001$, the full model fitted data better. Third, we used the product of coefficients approach to analyze moderated mediation effects. As illustrated in Figure S2, the interaction of adolescents' psychosocial flourishing and their authenticity positively predicted their CG intention ($b = 0.14$, $p < 0.001$, 95%CI [0.12, 0.17]), suggesting that the positive relation between adolescents' psychosocial flourishing and CG intention was moderated by their authenticity.

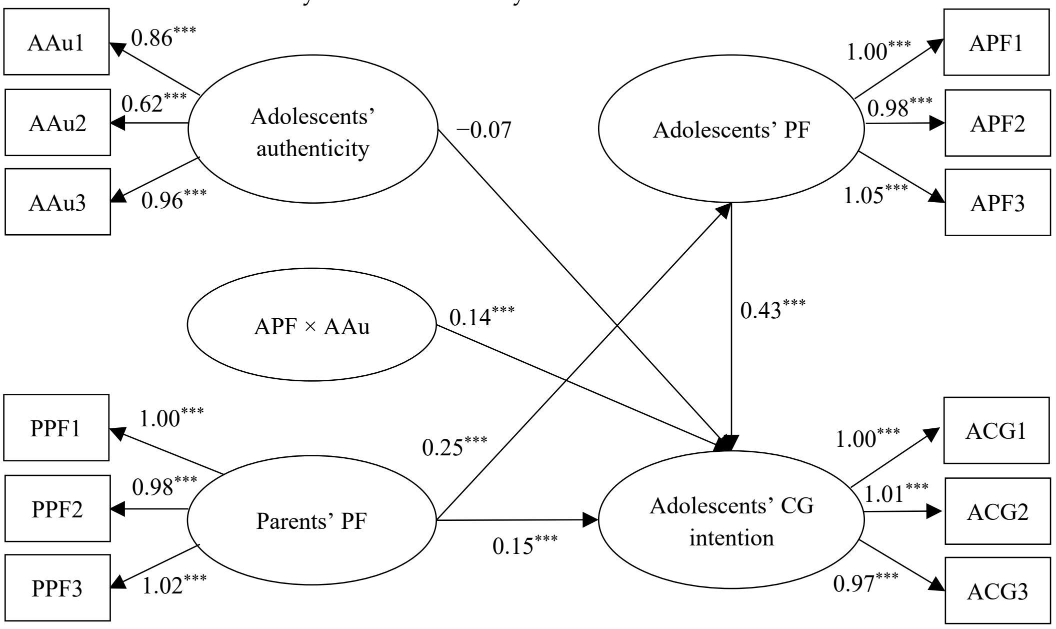

Figure S2. Moderated mediation model for Study 1. *Note.* Unstandardized coefficients are reported; PF = parents' psychosocial flourishing, APF = adolescents' psychosocial flourishing, AAu = adolescents' authenticity, CG = adolescents' charitable giving; PPF1-PPF3 = three parcels of parents' psychosocial flourishing; APF1-APF3 = three parcels of adolescents' psychosocial flourishing; AAu1-AAu3 = three parcels of adolescents' authenticity; ACG1-ACG3 = three parcels of adolescents' charitable giving intention. $^{***}p < 0.001$.

The follow-up simple slope analysis demonstrated that the positive association between adolescents' psychosocial flourishing and CG intention was significantly stronger among adolescents with high authenticity ($b_{simple} = 0.55$, $p < 0.001$, 95 % CI [0.48, 0.62]) than for adolescents with low authenticity ($b_{simple} = 0.30$, $p < 0.001$, 95 % CI [0.23, 0.36]).

The bias-corrected percentile bootstrap analyses further indicated that the indirect effect of parents' psychosocial flourishing on adolescents' CG intention through adolescents' psychosocial flourishing was moderated by adolescents' authenticity (index of moderated mediation = 0.04, $SE = 0.01$, 95% CI [0.02, 0.04]). The indirect relationship between parents' psychosocial flourishing and adolescents' CG intention was significantly stronger for participants with high authenticity ($b = 0.14$, $SE = 0.02$, 95 % CI [0.11, 0.15]), whereas this indirect relationship was weaker for participants with low authenticity ($b = 0.07$, $SE = 0.02$, 95 % CI [0.06, 0.08]). This pattern of results is consistent with the moderated mediation hypothesis, as the indirect effect of parents' psychosocial flourishing on adolescents' CG intention through adolescents' psychosocial flourishing was conditional on the level of adolescents' authenticity. Hypothesis 3 was supported.

Study 2

Measurement invariance

Longitudinal Measurement Invariance of Psychosocial Flourishing

Measurement invariance across the two waves was assessed using confirmatory factor analysis with MLR estimation. A unidimensional eight-item factor structure was specified at each time point, with corresponding residuals allowed to correlate. Scalar invariance was evaluated by comparing three nested models using the following criteria: ΔCFI ≥ −0.010 and ΔRMSEA ≤ 0.015, and ΔSRMR ≤ 0.030 for metric invariance and ΔSRMR ≤ 0.010 for scalar invariance (Chen, 2007; Cheung & Rensvold, 2002).

Configural model. The baseline configural model showed adequate fit to the data: $\chi^2(103) = 258.33$, scaling correction = 1.528, CFI = 0.946, RMSEA = 0.066 (90% CI [0.056, 0.076]), SRMR = 0.036, AIC = 16008.23. Standardized factor loadings ranged from 0.603 (APF04) to 0.813 (APF01) at T1 and from 0.868 (APF08) to 0.923 (APF05) at T2.

Metric model. Constraining the seven free factor loadings to equality across waves yielded the following fit: $\chi^2(110) = 283.45$, scaling correction = 1.497, CFI = 0.939, RMSEA = 0.067, SRMR = 0.055, AIC = 16023.66. Compared with the configural model, the changes in practical fit indices were negligible (ΔCFI = −0.007, ΔRMSEA = 0.001, ΔSRMR = 0.019), supporting metric invariance. The common standardized loadings in the metric model ranged from 0.719 to 0.789 at T1 and from 0.825 to 0.925 at T2.

Scalar model. Additional constraints on the eight item intercepts produced the following fit: $\chi^2(117) = 314.71$, scaling correction = 1.460, CFI = 0.931, RMSEA = 0.070, SRMR = 0.062, AIC = 16044.84. The fit deterioration relative to the metric model was minimal (ΔCFI = −0.008, ΔRMSEA = 0.003, ΔSRMR = 0.007), supporting full scalar invariance. The T1 latent mean was fixed at 0 for identification, and the T2 latent mean was estimated at −0.374 ($SE = 0.082$, $p < 0.001$; standardized mean difference = −0.235, $SE = 0.051$, $p < 0.001$), indicating a significant decrease in psychosocial flourishing over time. The latent correlation between waves was $r = 0.475$ ($p < 0.001$). In the scalar model, standardized loadings ranged from 0.672 to 0.787 at T1 and from 0.820 to 0.925 at T2.

Conclusion. Despite statistically significant chi-square difference tests (configural vs. metric: $\Delta\chi^2 = 24.34$, Δdf = 7, $p < 0.001$; metric vs. scalar: $\Delta\chi^2 = 35.52$, $\Delta df = 7$, $p < 0.001$), the practical fit indices clearly supported scalar longitudinal measurement invariance. All subsequent longitudinal analyses were therefore conducted on the assumption of comparable measurement structure across waves.

Longitudinal Measurement Invariance of CG Intention

Configural model. The baseline configural model exhibited acceptable model fit: $\chi^2(34) = 105.520$, scaling correction = 1.3926, CFI = 0.933, TLI = 0.912, RMSEA = 0.078 (90% CI [0.061, 0.095]), SRMR = 0.043, AIC = 8504.192. Standardized loadings ranged from 0.556 (T1CD05) to 0.783 (T1CD02) at T1 and from 0.753 (T3CD05) to 0.841 (T3CD04) at T3.

Metric model. Constraining all corresponding factor loadings to be equal across T1 and T3 generated the metric invariance model with adequate fit: $\chi^2(38) = 108.800$, scaling correction = 1.3789, CFI = 0.934, TLI = 0.922, RMSEA = 0.073 (90% CI [0.057, 0.090]), SRMR = 0.046, AIC = 8499.268. Compared with the configural model, the changes in practical fit indices were trivial and fully met the invariance criteria (ΔCFI = 0.001, ΔRMSEA = −0.005, ΔSRMR = 0.003), supporting metric invariance of CG intention over time. The standardized factor loadings under the metric invariance constraints ranged from 0.554 to 0.785 at T1 and from 0.753 to 0.834 at T3, all of which were statistically significant (ps < 0.001).

Scalar model (partial). Because the full scalar model with all intercepts constrained produced unacceptable fit deterioration, partial scalar invariance was tested. The intercepts of CD01 and CD03 were freely estimated across waves, whereas the intercepts of CD02, CD04, and CD05 were constrained to equality. The latent mean of CG_T1 was fixed at 0 for identification, and the latent mean of CG_T3 was freely estimated. The model showed acceptable fit: $\chi^2(40) = 118.762$, scaling correction = 1.3585, CFI = 0.927, TLI = 0.918, RMSEA = 0.075, SRMR = 0.053, AIC = 8506.576. Relative to the metric model, the fit changes were within acceptable limits (ΔCFI = −0.007, ΔRMSEA = 0.002, ΔSRMR = 0.007).

Conclusion. Although the chi-square values of the nested models increased significantly with stricter constraints, the practical fit index changes confirmed the longitudinal partial scalar measurement invariance of CG intention across T1 and T3 waves. The five-item scale of CG intention exhibited consistent factor structure and comparable psychometric properties over time. Therefore, longitudinal analyses on the developmental changes and cross-time relationships of CG intention in subsequent studies were statistically reliable.

Table S3. Longitudinal Measurement Invariance Across Waves

Construct	Model	χ^2	df	sc	CFI	RMSEA	SRMR	$\Delta\chi^2$	Δdf	p	ΔCFI	ΔRMSEA	ΔSRMR
Psychosocial Flourishing	Configural	258.33	103	1.528	.946	.066	.036	—	—	—	—	—	—
	Metric	283.45	110	1.497	.939	.067	.055	24.34	7	<.001	−.007	.001	.019
	Scalar (Full)	314.71	117	1.460	.931	.070	.062	35.52	7	<.001	−.008	.003	.007
CG Intention	Configural	105.52	34	1.393	.933	.078	.043	—	—	—	—	—	—
	Metric	108.80	38	1.379	.934	.073	.046	3.28	4	.511	.001	−.005	.003
	Scalar (Partial)	118.76	40	1.359	.927	.075	.053	9.96	2	.007	−.007	.002	.007

Note. $\Delta\chi^2$ values are Satorra–Bentler scaled chi-square differences. For psychosocial flourishing, the scalar model constrained all intercepts with the T2 latent mean freely estimated. For CG intention, partial scalar invariance was achieved by constraining the intercepts of CD02, CD04, and CD05 to equality across waves while freely estimating the intercepts of CD01 and CD03.

Table S4. Cross-Reporter Measurement Invariance (Parent vs. Adolescent) at T1

Study	Model	CFI	RMSEA	SRMR	ΔCFI	ΔRMSEA	ΔSRMR
Study 1	Configural	.969	.054	.031	—	—	—
	Metric	.966	.053	.058	−.003	−.001	.027
	Scalar	.959	.054	.062	−.007	.001	.004
Study 2	Configural	.952	.070	.036	—	—	—
	Metric	.949	.068	.049	−.003	−.002	.013
	Scalar	.944	.067	.053	−.005	−.001	.004

Study 2 Primary Model – Longitudinal Mediation SEM Syntax:

```
TITLE: Longitudinal mediation model
DATA:
   FILE = Charity.dat;
VARIABLE:
   NAMES = Agender Aage Pgender Page Fedu Medu Fincome
                          T1APF T1ACG x1-x3 m1-m3 z1-z3 y1-y3 class;
   USEVARIABLE = Agender Aage Pgender Page Fedu Medu Fincome
                         T1APF T1ACG x1-x3 m1-m3 y1-y3;
   MISSING = ALL(-999);
ANALYSIS:
   ESTIMATOR = ML;
   BOOTSTRAP = 5000;
MODEL:
   ! Latent factor definitions
   T1PPF BY x1-x3*;
   T2APF BY m1-m3*;
   T3ACG BY y1-y3*;
   T1PPF@1; T2APF@1; T3ACG@1;
   ! Autoregressive paths (baseline controls)
   T2APF ON T1APF;           ! T1 APF -> T2 APF
   T3ACG ON T1ACG;           ! T1 ACG -> T3 ACG
   ! Core longitudinal paths
   T2APF ON T1PPF;             ! H1
   T3ACG ON T2APF;             ! H2: mediator path
   T3ACG ON T1PPF;             ! H2: direct effect
   ! Covariate paths
   T3ACG ON Agender Aage Pgender Page Fedu Medu Fincome;
MODEL INDIRECT:
   T3ACG IND T2APF T1PPF;
OUTPUT:
   STANDARDIZED CINTERVAL(BCBOOTSTRAP);
```

Study 2 Primary Model – Longitudinal Moderated Mediation LSM Syntax:

```
TITLE: Longitudinal LMS full model
DATA:
   FILE = Charity.dat;
VARIABLE:
   NAMES = Agender Aage Pgender Page Fedu Medu Fincome
                     T1APF T1ACG x1-x3 m1-m3 z1-z3 y1-y3 class;
   USEVARIABLE = Agender Aage Pgender Page Fedu Medu Fincome
                  T1APF T1ACG x1-x3 m1-m3 z1-z3 y1-y3;
   MISSING = ALL(-999);
ANALYSIS:
   TYPE = RANDOM;
   ESTIMATOR = ML;
   ALGORITHM = INTEGRATION;
   BOOTSTRAP = 5000;
   PROCESSORS = 8;
   CONVERGENCE = 0.1;
MODEL:
   ! Latent factor definitions (aligned with baseline)
   T1PPF   BY x1-x3;
   T2APF   BY m1-m3;
   T2AAU   BY z1-z3*;
   T2AAU@1;
   T3ACG   BY y1-y3;
   ! Latent interaction term
   MZ | T2APF XWITH T2AAU;
   ! Core longitudinal paths (labeled for MODEL CONSTRAINT)
   T2APF   ON T1PPF(a1);
   T3ACG   ON T2APF(b1);
   T3ACG   ON T2AAU(b2);
   T3ACG   ON MZ(b3);
   T3ACG   ON T1PPF(c);
   ! Autoregressive controls (from baseline)
   T2APF   ON T1APF;
   T3ACG   ON T1ACG;
   ! Covariate controls on both endogenous factors (from baseline)
   T3ACG   ON Agender Aage Pgender Page Fedu Medu Fincome;
   ! Latent residual covariance between concurrent T2 constructs (from baseline)
   T2AAU WITH T2APF;
MODEL CONSTRAINT:
   NEW(LOW_AAU MED_AAU HIGH_AAU
       IND_LOWAAU IND_MEDAAU IND_HIAAU
       IMAPF
```

```
        TOT_LOWAAU TOT_MEDAAU TOT_HIAAU);
   LOW_AAU = -1;
   MED_AAU = 0;
   HIGH_AAU = 1;
   IND_LOWAAU = a1*b1 + a1*b3*LOW_AAU;
   IND_MEDAAU = a1*b1 + a1*b3*MED_AAU;
   IND_HIAAU = a1*b1 + a1*b3*HIGH_AAU;
   IMAPF = a1*b3;
   TOT_LOWAAU = IND_LOWAAU + c;
   TOT_MEDAAU = IND_MEDAAU + c;
   TOT_HIAAU = IND_HIAAU + c;
   PLOT(LOMOD MEDMOD HIMOD);
   LOOP(XVAL,-3,3,0.1);
   LOMOD = IND_LOWAAU*XVAL;
   MEDMOD = IND_MEDAAU*XVAL;
   HIMOD = IND_HIAAU*XVAL;
PLOT:
   TYPE = PLOT2;
OUTPUT:
   CINTERVAL(BCBOOTSTRAP);
```

OUTPUT:

MODEL RESULTS

	Estimate	S.E.	Est./S.E.	Two-Tailed P-Value
T1PPF BY				
X1	1.000	0.000	999.000	999.000
X2	0.976	0.036	27.135	0.000
X3	0.908	0.042	21.501	0.000
T2APF BY				
M1	1.000	0.000	999.000	999.000
M2	1.013	0.030	34.278	0.000
M3	1.014	0.029	34.463	0.000
T2AAU BY				
Z1	1.034	0.069	15.001	0.000
Z2	1.048	0.059	17.610	0.000
Z3	1.164	0.053	21.939	0.000
T3ACG BY				
Y1	1.000	0.000	999.000	999.000
Y2	1.152	0.070	16.422	0.000
Y3	1.126	0.072	15.736	0.000
T2APF ON				
T1PPF	0.251	0.081	3.091	0.002
T3ACG ON				

T2APF	0.142	0.035	3.998	0.000
T2AAU	-0.142	0.061	-2.347	0.019
MZ	0.064	0.022	2.879	0.004
T1PPF	0.117	0.051	2.280	0.023
T2APF ON				
T1APF	0.482	0.080	6.011	0.000
T3ACG ON				
T1ACG	0.322	0.072	4.453	0.000
AGENDER	0.107	0.087	1.230	0.219
AAGE	-0.057	0.051	-1.119	0.263
PGENDER	-0.237	0.109	-2.174	0.030
PAGE	-0.004	0.008	-0.471	0.638
FEDU	-0.092	0.096	-0.952	0.341
MEDU	0.109	0.087	1.256	0.209
FINCOME	0.060	0.049	1.221	0.222
T2AAU WITH				
T2APF	0.605	0.088	6.861	0.000
T1PPF	0.097	0.060	1.607	0.108
Intercepts				
X1	5.175	0.058	89.593	0.000
X2	5.324	0.053	100.681	0.000
X3	5.148	0.063	81.600	0.000
M1	2.144	0.408	5.256	0.000
M2	2.139	0.428	4.999	0.000
M3	2.094	0.416	5.033	0.000
Z1	4.203	0.062	67.898	0.000
Z2	3.855	0.061	62.755	0.000
Z3	3.947	0.062	63.987	0.000
Y1	2.945	0.952	3.094	0.002
Y2	2.763	1.113	2.483	0.013
Y3	2.788	1.079	2.583	0.010
Variances				
T1PPF	1.006	0.075	13.382	0.000
T2AAU	1.000	0.000	999.000	999.000
Residual Variances				
X1	0.218	0.044	4.934	0.000
X2	0.168	0.036	4.718	0.000
X3	0.412	0.059	6.959	0.000
M1	0.214	0.055	3.894	0.000
M2	0.312	0.057	5.424	0.000
M3	0.242	0.052	4.622	0.000
Z1	0.374	0.052	7.204	0.000
Z2	0.394	0.048	8.214	0.000
Z3	0.194	0.045	4.318	0.000

Y1	0.342	0.054	6.375	0.000
Y2	0.095	0.029	3.288	0.001
Y3	0.355	0.059	5.999	0.000
T2APF	1.734	0.156	11.105	0.000
T3ACG	0.462	0.058	7.948	0.000
New/Additional Parameters				
LOW_AAU	-1.000	0.000	*********	0.000
MED_AAU	0.000	0.000	0.000	1.000
HIGH_AAU	1.000	0.000	*********	0.000
IND_LOWA	0.019	0.013	1.551	0.121
IND_MEDA	0.036	0.015	2.313	0.021
IND_HIAA	0.052	0.021	2.434	0.015
IMAPF	0.016	0.008	1.955	0.051
TOT_LOWA	0.136	0.050	2.703	0.007
TOT_MEDA	0.152	0.051	2.977	0.003
TOT_HIAA	0.168	0.053	3.166	0.002

CONFIDENCE INTERVALS OF MODEL RESULTS

	Lower .5%	Lower 2.5%	Lower 5%	Estimate	Upper 5%	Upper 2.5%	Upper .5%
T1PPF BY							
X1	1.000	1.000	1.000	1.000	1.000	1.000	1.000
X2	0.907	0.909	0.931	0.976	1.055	1.061	1.080
X3	0.787	0.787	0.821	0.908	0.965	0.986	1.000
T2APF BY							
M1	1.000	1.000	1.000	1.000	1.000	1.000	1.000
M2	0.947	0.966	0.979	1.013	1.076	1.087	1.110
M3	0.937	0.957	0.974	1.014	1.067	1.079	1.110
T2AAU BY							
Z1	0.875	0.884	0.915	1.034	1.134	1.163	1.180
Z2	0.924	0.930	0.951	1.048	1.139	1.162	1.173
Z3	1.040	1.059	1.075	1.164	1.246	1.256	1.279
T3ACG BY							
Y1	1.000	1.000	1.000	1.000	1.000	1.000	1.000
Y2	1.032	1.032	1.036	1.152	1.250	1.275	1.365
Y3	0.977	0.981	0.995	1.126	1.241	1.278	1.317
T2APF ON							
T1PPF	0.023	0.051	0.107	0.251	0.379	0.412	0.434
T3ACG ON							
T2APF	0.057	0.073	0.075	0.142	0.199	0.210	0.219
T2AAU	-0.317	-0.317	-0.258	-0.142	-0.065	-0.053	-0.041
MZ	0.007	0.020	0.030	0.064	0.108	0.111	0.125
T1PPF	0.013	0.030	0.051	0.117	0.217	0.240	0.269
T2APF ON							
T1APF	0.284	0.311	0.331	0.482	0.594	0.599	0.660

T3ACG ON							
T1ACG	0.165	0.183	0.214	0.322	0.443	0.478	0.537
AGENDER	-0.109	-0.109	-0.049	0.107	0.255	0.270	0.290
AAGE	-0.183	-0.183	-0.183	-0.057	0.004	0.013	0.032
PGENDER	-0.529	-0.455	-0.446	-0.237	-0.071	-0.030	0.017
PAGE	-0.022	-0.020	-0.018	-0.004	0.010	0.011	0.014
FEDU	-0.299	-0.299	-0.294	-0.092	0.029	0.068	0.161
MEDU	-0.157	-0.026	0.012	0.109	0.248	0.250	0.263
FINCOME	-0.038	-0.038	-0.029	0.060	0.130	0.147	0.164
T2AAU WITH							
T2APF	0.372	0.421	0.444	0.605	0.748	0.757	0.832
T1PPF	-0.048	-0.045	-0.011	0.097	0.194	0.211	0.240
Intercepts							
X1	5.067	5.067	5.073	5.175	5.256	5.271	5.287
X2	5.172	5.222	5.232	5.324	5.418	5.430	5.442
X3	4.974	4.999	5.000	5.148	5.235	5.241	5.297
M1	1.177	1.411	1.557	2.144	2.835	2.927	3.136
M2	1.000	1.452	1.502	2.139	2.847	3.031	3.186
M3	1.030	1.446	1.510	2.094	2.796	2.923	3.010
Z1	4.045	4.045	4.053	4.203	4.276	4.290	4.329
Z2	3.705	3.705	3.705	3.855	3.927	3.942	3.968
Z3	3.793	3.793	3.793	3.947	4.014	4.022	4.064
Y1	0.987	1.159	1.494	2.945	4.257	5.175	5.225
Y2	0.419	1.004	1.119	2.763	4.580	5.396	5.604
Y3	0.549	0.569	1.069	2.788	4.209	4.750	5.392
Variances							
T1PPF	0.812	0.865	0.881	1.006	1.133	1.135	1.190
T2AAU	1.000	1.000	1.000	1.000	1.000	1.000	1.000
Residual Variances							
X1	0.122	0.143	0.147	0.218	0.291	0.302	0.327
X2	0.070	0.097	0.100	0.168	0.221	0.249	0.249
X3	0.279	0.326	0.341	0.412	0.549	0.563	0.612
M1	0.110	0.139	0.150	0.214	0.371	0.371	0.387
M2	0.187	0.223	0.241	0.312	0.422	0.422	0.427
M3	0.145	0.153	0.158	0.242	0.339	0.348	0.356
Z1	0.247	0.255	0.278	0.374	0.443	0.468	0.496
Z2	0.308	0.317	0.326	0.394	0.502	0.502	0.503
Z3	0.087	0.133	0.140	0.194	0.296	0.296	0.315
Y1	0.200	0.239	0.243	0.342	0.425	0.444	0.458
Y2	0.003	0.041	0.044	0.095	0.143	0.155	0.167
Y3	0.224	0.238	0.252	0.355	0.448	0.466	0.501
T2APF	1.316	1.430	1.495	1.734	1.973	2.034	2.087
T3ACG	0.352	0.366	0.383	0.462	0.591	0.591	0.591

New/Additional Parameters

LOW_AAU	-1.000	-1.000	-1.000	-1.000	-1.000	-1.000	-1.000
MED_AAU	0.000	0.000	0.000	0.000	0.000	0.000	0.000
HIGH_AAU	1.000	1.000	1.000	1.000	1.000	1.000	1.000
IND_LOWA	-0.002	0.000	0.002	0.019	0.042	0.045	0.054
IND_MEDA	0.003	0.009	0.012	0.036	0.063	0.065	0.073
IND_HIAA	0.004	0.013	0.019	0.052	0.090	0.091	0.103
IMAPF	0.001	0.004	0.007	0.016	0.034	0.038	0.040
TOT_LOWA	0.034	0.038	0.046	0.136	0.214	0.239	0.273
TOT_MEDA	0.045	0.057	0.077	0.152	0.239	0.278	0.280
TOT_HIAA	0.049	0.065	0.092	0.168	0.259	0.282	0.296

<u>All longitudinal SEM and LMS models in Study 2 were re-estimated using cluster-robust standard errors. Results of the cluster-robust sensitivity analyses are reported below:</u>

Testing for Longitudinal Intergenerational Association in Psychosocial Flourishing

As in Study 1, we first established a simple regression model with latent variables to test the effect of parents' psychosocial flourishing at T1 on adolescents' psychosocial flourishing at T2, using TYPE = COMPLEX to account for the nesting of dyads within classrooms and 5,000 bootstrap draws for bias-corrected confidence intervals. After controlling for adolescents' gender, age, parents' gender, age, father's education, mother's education, family monthly income, and adolescents' psychosocial flourishing at T1, parents' psychosocial flourishing at T1 was positively associated with subsequent adolescents' psychosocial flourishing at T2 (b = 0.19, $p < 0.001$, 95%CI [0.13, 0.26]). Hypothesis 1 was supported.

Testing for Longitudinal Mediation Effect

A simple regression model with latent variables was then estimated to test the direct effect of parents' psychosocial flourishing at T1 on adolescents' CG intention at T3. Results showed that parents' psychosocial flourishing at T1 was positively related to adolescents' CG intention at T3 (b = 0.21, p = 0.001, 95%CI [0.11, 0.32]).

As illustrated in Figure S3, after controlling for adolescents' gender, age, parents' gender, age, father's education, mother's education, family monthly income, adolescents' psychosocial flourishing and CG intention at T1, the effect of parents' psychosocial flourishing at T1 on adolescents' psychosocial flourishing at T2 was significant (b = 0.18, $p < 0.001$, 95%CI [0.10, 0.25]). When adolescents' psychosocial flourishing at T2 was included as a mediator, it was positively associated with their CG intention at T3 (b = 0.24, p = 0.001, 95%CI [0.13, 0.37]), and the direct effect of parents' psychosocial flourishing at T1 on adolescents' CG intention at T3 decreased accordingly (b = 0.16, p = 0.02, 95%CI [0.05, 0.28]). Bootstrapping results are again consistent with Hypothesis 2 that adolescents' psychosocial flourishing at T2 mediated the longitudinal association between parents' psychosocial flourishing at T1 and adolescents' CG intention at T3 ($b_{indirect}$ = 0.04, SE = 0.02, p = 0.01, R^2 = 0.24, 95%CI [0.03, 0.13]). The mediating effect accounted for 20.39% of the total effect of parents' psychosocial flourishing at T1 on adolescents' CG intention at T3.

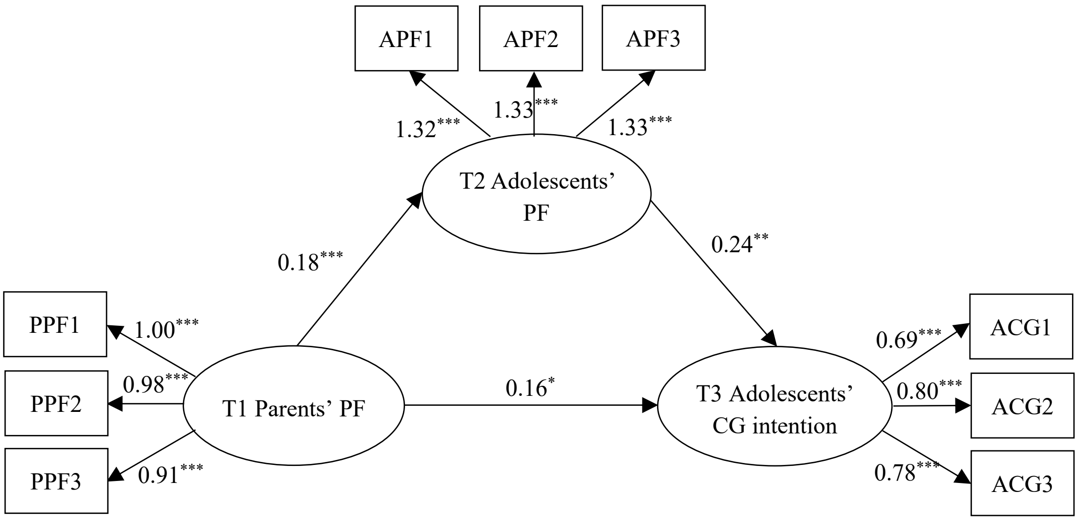

Figure S3. Mediation model for Study 2. *Note.* Unstandardized coefficients are reported; PF = parents' psychosocial flourishing, CG = charitable giving; PPF1-PPF3 = three parcels of parents' psychosocial flourishing; APF1-APF3 = three parcels of adolescents' psychosocial flourishing; ACG1-ACG3 = three parcels of adolescents' charitable giving intention. $^{*}p < 0.05$; $^{***}p < 0.001$.

Testing for Longitudinal Moderated Mediation

The LMS analysis was conducted to examine the moderating effect of adolescents' authenticity at T2. We first tested a baseline model which excludes the latent interaction term (i.e., adolescents' psychosocial flourishing at T2 × adolescents' authenticity at T2), the results showed that the model fitted data well: $\chi^2 = 250.116$, $df = 147$, $p < 0.001$, CFI = 0.97, TLI = 0.96, RMSEA = 0.045, SRMR = 0.068. Then, we tested whether the model fit of the full model (i.e., the moderated mediation model including the latent interaction term) is significantly better than that of the baseline model. The value of AIC_{full} (9873.380) is less than $AIC_{baseline}$ (9879.440). Furthermore, as indicated by the results of the log-likelihood ratio test, $\chi^2 = -2[(-4888.720) - (-4884.690)] = 8.06$, $df = 1$, $p = 0.005$, the full model fitted data better. As illustrated in Figure S4, the interaction of adolescents' psychosocial flourishing at T2 and adolescents' authenticity at T2 positively predicted adolescents' CG intention at T3 ($b = 0.06$, $p = 0.01$, 95%CI [0.03, 0.10]), indicating that adolescents' authenticity at T2 moderated the association between adolescents' psychosocial flourishing at T2 and their CG intention at T3.

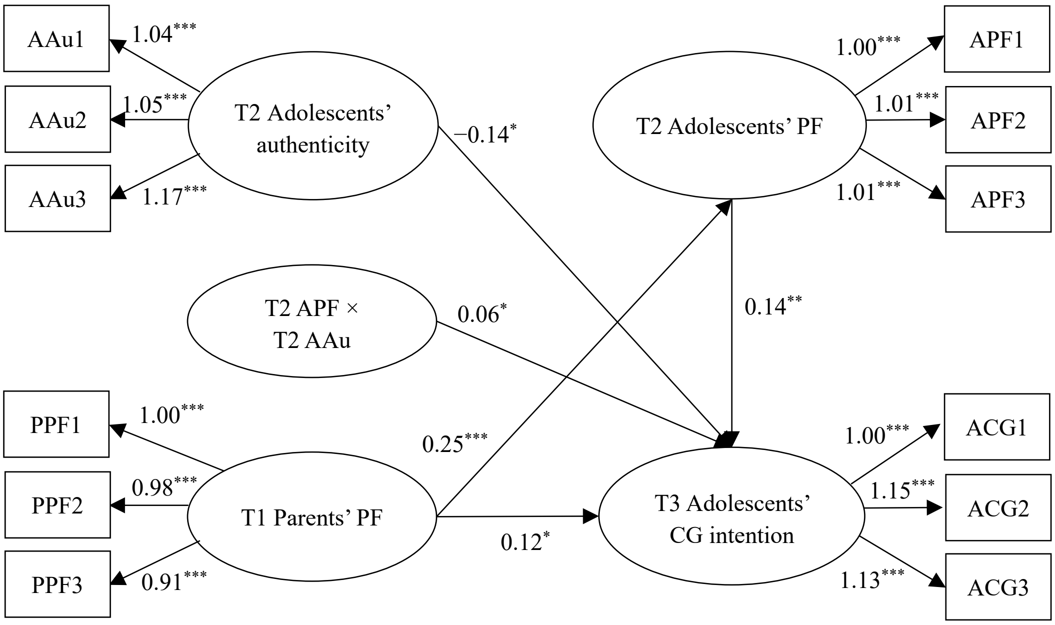

Figure S4. Moderated mediation model for Study 2. *Note.* Unstandardized coefficients are reported; PF = parents' psychosocial flourishing, APF = adolescents' psychosocial flourishing, AAu = adolescents' authenticity, CG = adolescents' charitable giving; PPF1-PPF3 = three parcels of parents' psychosocial flourishing; APF1-APF3 = three parcels of adolescents' psychosocial flourishing; AAu1-AAu3 = three parcels of adolescents' authenticity; ACG1-ACG3 = three parcels of adolescents' charitable giving intention. $^{**}p < 0.01$; $^{***}p < 0.001$.

The follow-up simple slope analysis showed that the link between adolescents' psychosocial flourishing at T2 and their CG intention at T3 was significantly stronger for adolescents with high authenticity ($b_{simple} = 0.31$, $p < 0.001$, 95 % CI [0.23, 0.40]) than for adolescents with low authenticity ($b_{simple} = 0.14$, $p = 0.001$, 95 % CI [0.07, 0.21]).

The bias-corrected percentile bootstrap analyses further supported that the indirect effect of parents' psychosocial flourishing at T1 on adolescents' CG intention at T3 through adolescents' psychosocial flourishing at T2 was moderated by adolescents' authenticity at T2 (index of moderated mediation = 0.02, *SE* = 0.01, 95% CI [0.01, 0.04]). The indirect relationship between parents' psychosocial flourishing at T1 and adolescents' CG intention at T3 was significantly stronger for participants with high authenticity at T2 ($b = 0.05$, $SE = 0.02$, 95 % CI [0.03, 0.09]), whereas this indirect relationship was weaker for participants with low authenticity at T2 ($b = 0.02$, $SE = 0.01$, 95 % CI [0.00, 0.04]). This pattern of results is consistent with the moderated mediation hypothesis, as the indirect effect of parents' psychosocial flourishing at T1 on adolescents' CG intention at T3 through adolescents' psychosocial flourishing at T2 was conditional on the level of adolescents' authenticity at T2. Hypothesis 3 was supported.
